# Chemical Bonding by the Chemical Orthogonal Space of Reactivity

**DOI:** 10.3390/ijms22010223

**Published:** 2020-12-28

**Authors:** Mihai V. Putz

**Affiliations:** 1 Laboratory of Structural and Computational Physical-Chemistry for Nanosciences and QSAR, Biology-Chemistry Department, Faculty of Chemistry, Biology, Geography, West University of Timisoara, Pestalozzi Street No. 16, RO-300115 Timisoara, Romania; mihai.putz@e-uvt.ro or mv_putz@yahoo.com; 2 Laboratory of Renewable Energies 1—Scientific Research, R&D National Institute for Electrochemistry and Condensed Matter, Street Dr. Aurel Paunescu Podeanu No. 144, RO-300569 Timisoara, Romania

**Keywords:** electronegativity, chemical hardness, chemical orthogonal space of reactivity, chemical power index, charge transfer, chemical orthogonal space, Parr–Pearson bonding model

## Abstract

The fashionable Parr–Pearson (PP) atoms-in-molecule/bonding (AIM/AIB) approach for determining the exchanged charge necessary for acquiring an equalized electronegativity within a chemical bond is refined and generalized here by introducing the concepts of chemical power within the chemical orthogonal space (COS) in terms of electronegativity and chemical hardness. Electronegativity and chemical hardness are conceptually orthogonal, since there are opposite tendencies in bonding, i.e., reactivity vs. stability or the HOMO-LUMO middy level vs. the HOMO-LUMO interval (gap). Thus, atoms-in-molecule/bond electronegativity and chemical hardness are provided for in orthogonal space (COS), along with a generalized analytical expression of the exchanged electrons in bonding. Moreover, the present formalism surpasses the earlier Parr–Pearson limitation to the context of hetero-bonding molecules so as to also include the important case of covalent homo-bonding. The connections of the present COS analysis with PP formalism is analytically revealed, while a numerical illustration regarding the patterning and fragmentation of chemical benchmarking bondings is also presented and fundamental open questions are critically discussed.

## 1. Introduction

Electronegativity (*χ*) and chemical hardness (*η*) may be considered the “great arcanes” of modern conceptual chemistry. This is because they seem to be related with whatever classic and quantum concepts chemistry develops or encompasses [1,2,3].

Their definitions closely relate to the total or valence energy (*E*) variation respecting with the total/valence number of electrons (*N*) exchanged/involved in an interaction or for achieving physical-chemical state equilibrium in a given external (atomic, molecular, slid state, etc.) potential (*V*(**r**)), eventually expressed spectroscopically by adiabatic HOMO and LUMO highest occupied and lowest unoccupied molecular orbitals, respectively, as reflected by the modern density functional theory [4,5,6]

(1) $$\chi =-{\left(\frac{\partial E}{\partial N}\right)}_{V(r)}≅\frac{\varepsilon_{LUMO}+\varepsilon_{HOMO}}{2}$$

(2) $$\eta =-\frac{1}{2}{\left(\frac{\partial \chi }{\partial N}\right)}_{V(r)}≅\frac{\varepsilon_{LUMO}-\varepsilon_{HOMO}}{2}$$

They may be regarded as the chemical counterpart for the physical velocity and acceleration [7], since the electronegativity actually expresses the energy kinetics on the electrons’ exchange regarded as a coordinate [8]. The same stands for the chemical hardness which associates with the inertial force to such behavior [9,10] and in accordance with the working Equations (1) and (2). When the electrons’ exchange is restrained to the orbital occupancy [11,12], i.e., between 0 and 2 electrons, this case covers also the chemical bonding phenomenology. Electronegativity and chemical hardness may be also described by the creation and annihilation quantum operators [13], thus achieving a certain degree of observability [14]. Moreover, on the electronegativity side, it is explicitly stated as the negative pair of chemical potential of the system [15], while chemical hardness is more related to the instabilities (indeterminacies) of the chemical systems [16]. They also support, via Koopmans’ theorem of frozen frontier orbitals upon the electronic exchange [17], the finite difference relationship with the ionization and electronic affinity potentials, thus appearing with numerical/spectroscopic predictive or quantitative explicative character [18]. Due to the analytic expressions of Equations (1) and (2), electronegativity and chemical hardness may be further expanded in terms of energy [19] and on a number of density functional levels of approximation for the total/valence energy, as available [20]. The Parr–Pearson approach [4,5] considers the expansion of the atomic energy of an atom *A* around its neutral state (up to the second order) aiming achieving an atom-in-bonding status through the charge transfer.

(3) $$E_{A}(N_{\langle A\rangle })≅E_{A}(N_{A})+{\left(\frac{\partial E_{A}}{\partial N_{\langle A\rangle }}\right)}_{0}(N_{\langle A\rangle }-N_{A})+\frac{1}{2}{\left(\frac{\partial^{2}E_{A}}{\partial N_{\langle A\rangle }^{2}}\right)}_{0}{\left(N_{\langle A\rangle }-N_{A}\right)}^{2}\;\equiv E_{A}(N_{A})-\chi_{A}\Delta N+\eta_{A}{\left(\Delta N\right)}^{2}$$

Here, the electronegativity ($\chi_{A}$) and chemical hardness ($\eta_{A}$) have assumed with definitions (1) and (2) as above. However, according with Equation (1), the electronegativity of the atom *A* in a certain bonding unfolds as

(4) $$\chi_{\langle A\rangle }=-\frac{\partial E_{A}}{\partial N_{\langle A\rangle }}=\chi_{A}-2\eta_{A}\Delta N$$

Let us now consider the formation of a diatomic molecule *AB* acquiring the equilibrium at the separating distance $R_{AB}$. For an infinitesimal transfer of electronic charges between the bonding’s atoms, i.e., $N_{\langle A\rangle }=N_{A}-dN$ and $N_{\langle B\rangle }=N_{B}+dN$, the variation in the total energy $E=E_{A}+E_{B}$ can be written as

(5) $$dE={\left(\frac{\partial E}{\partial N_{\langle A\rangle }}\right)}_{N_{B},R_{AB}}(N_{\langle A\rangle }-N_{A})+{\left(\frac{\partial E}{\partial N_{\langle B\rangle }}\right)}_{N_{A},R_{AB}}(N_{\langle B\rangle }-N_{B})+{\left(\frac{\partial E}{\partial R_{AB}}\right)}_{N_{A},N_{B}}dR_{AB}$$

On the other side, in the fundamental equilibrium state, one has the customary variational conditions fulfilled—that is $\partial E/\partial R_{AB}=0,dE=0$, so that Equation (5) is reduced to the following form:

(6) $${\left(\frac{\partial E}{\partial N_{\langle A\rangle }}\right)}_{N_{B},R_{AB}}={\left(\frac{\partial E}{\partial N_{\langle B\rangle }}\right)}_{N_{A},R_{AB}}$$

Equation (6) corresponds to the naturally recovered principle of equality of the atoms’ electronegativities in bonding.

Further on, the application of principle (6) to the atoms *A* and *B* in *AB* molecule yields Equation (7).

(7) $$\chi_{\langle A\rangle }=\chi_{A}+2\eta_{A}\Delta N=\chi_{\langle B\rangle }=\chi_{B}-2\eta_{B}\Delta N=\chi_{AB}$$

The charge transfer turns from Equation (7) into

(8) $$\Delta N=\frac{\chi_{B}-\chi_{A}}{2(\eta_{A}+\eta_{B})}$$

The charge transfer is, nevertheless, accompanied by the released energy, written as

(9) $$\Delta E==-\frac{1}{4}\frac{{\left(\chi_{B}-\chi_{A}\right)}^{2}}{\left(\eta_{A}+\eta_{B}\right)}$$

The average of atomic’ electronegativity in bonding can be obtained through combining Equations (7) and (8) within the expression

(10) $$\chi_{AB}\equiv \bar{\chi }=\frac{\eta_{A}\chi_{B}+\eta_{B}\chi_{A}}{\eta_{A}+\eta_{B}}$$

However, the Parr–Pearson picture does not provide a direct evaluation averaged hardness in bonding; however, a companion formulation can be laid down from relation (10) once the proportionality between electronegativity and hardness is assumed at both atomic and bonding levels in terms of a sort of “universal” factor (γ) [11].

(11) $$\chi_{A}=\gamma \;\eta_{A}\;\chi_{B}=\gamma \;\eta_{B}\;\chi_{AB}=\gamma \;\eta_{AB}$$

Now, it is obvious that, through inserting relations (11) back in (10), the average for hardness in bonding simply results as

(12) $$\eta_{AB}\equiv \bar{\eta }=2\frac{\eta_{A}\eta_{B}}{\eta_{A}+\eta_{B}}$$

It is worth noting that the assumption made in (11) claims that all electronegativity-hardness pairs are correlated by the same factor γ, either for atoms or molecules.

The Parr–Pearson picture of atoms in bonding (AIB) comes in the first place due to a limitation in energy expansion, restricted only to the charge exchanges in Equation (3) without caring about the potential effects [4,5]. Moreover, this treatment seems not to correlate the electronegativity average $\bar{\chi }$ with the chemical hardness average $\bar{\eta }$ in a consistent manner. Further potential expansions of the Parr–Pearson model were accomplished [19], but the raised difficulties concerning the correlation between $\bar{\chi }$ and $\bar{\eta }$ still remain.

Nevertheless, the interest of chemical reactivity indices is continuously high due to their conceptual and applicative use, namely because

- Electronegativity and chemical hardness actually furnish the most general principles of chemistry, due to their variational character [21,22], thus supporting both the equilibrium as well as the equilibrium fluctuation of isolated and interacting states, as reflected in the celebrated principles of equalization electronegativity, minimum electronegativity, maximum hardness, and hard and soft acids and bases [23,24,25,26,27];
- The atomic scales computed with electronegativity and chemical hardness generally parallel the other structural parameters’ dependencies, such as estimated by atomic radii [28], ionization potential [29], electronic affinity [30], diamagnetic susceptibility [31], polarizability [32], etc. Thus, they prove once more their structural and eventually observational features, despite the “many faces” of their appearance [33,34,35,36,37,38];
- The molecular use of electronegativity and chemical hardness are equally valuable: it spans from modeling the chemical bonding by HOMO-LUMO coupling through the application of their chemical reactivity principles (i.e., the above variational principles) [39,40,41], to the localization-delocalization of electronic characterization and bond sharing of atoms in molecules [42]. The purpose is to model another specific chemical concept as the aromaticity [43], to the use of their intriguing parabolic combination in the total/valence energy to provide the molecular reactivity hierarchies [44], to model the toxicity and the eco-, bio-, and pharmacological activities by the consecrated quantitative structure-activity relationships (QSARs), as well by the recent Quantum-SAR (Qua-SARs) [45,46]. It may also include the recent coloring framework of the topological approaches offered by chemical graph theory [47] either by discrimination on chemical bonding path in molecules, or by chemical bonding in adjacencies [48], this way succeeding in discriminating for the chemical conjugation and providing the unified treatment of kenograms and pleograms [49], while offering new development space in chemical graph theory [50];
- Electronegativity and chemical hardness are also used as the benchmark qualitative framework in providing the oxidation and reduction potentials in electrochemistry models and in the design of efficient electro-photovoltaic cells, with great insight in selecting the appropriate sensitizer on anodic electrode, especially in the current modeling of the third generation of photosensitizes based on quantum-dots technology [51,52].

All in all, it is clear that electronegativity and chemical hardness offer the fundamental as well as the applicative tools for treating the basics of the chemical principles as well as their synergy with other physical processes. However, they will be employed in the present endeavor for generalizing the description of binding of atoms in molecules from simple energetic expansion coupled with the equalization principle of the atoms’ electronegativities in molecule, as according to Parr and Pearson [5], to the actual picture based on the chemical orthogonal space (COS) of electronegativity and chemical hardness (*χ*, *η*) [53]. Actually, this approach seems natural and necessary while assuming the two chemical reactivity indices as the complementary indices, so completing the (almost) entire space of chemical reactivity, qualitatively by their principles and quantitative by their Equations (1) and (2). Moreover, since the present approach also contributes to unifying these chemical concepts and their principles in a phenomenological orthogonal space, this is consistent with any physical quantum or algebraic (this way universal) description of the chemical interaction in general. We may refer to the recent studies on Qua-SAR(*χ*, *η*), ref. [3] (Vol. 5), and especially on chemical bonding, ref. [3] (Vol. 3).

Along with these approaches, the present work puts forward an original view on COS extension of the Parr–Pearson charge transfer, on the allied electronegativity and chemical hardness atoms-in-bonding, with two major objectives:

(**1-COS**) The introduced parameter, chemical power, is defined and developed into a figure of merit for evaluating chemical bonding with better correlation of electronegativity and chemical hardness principles respecting the Parr–Pearson model; and

(**2-COS**) Arriving to the average hardness by a means of the parameter introduced in (1-COS), within the actual COS model, so fulfilling also the better correlation than with PP approach respecting chemical reactivity principles in chemical bonds and bonding.

The COS model viability would be justified a posteriori by its applicability to the homonuclear as well as the heteronuclear bonding. Moreover, the current theory (i.e., COS) comprises the classical Parr–Pearson (PP) expression in certain analytical condition. To achieve this, the paper is divided into the following two parts:

- Section 2 introduces the chemical power concept in the chemical orthogonal space this way conceptually and analytically linking the electronegativity and chemical hardness by their ratio, considering it as the precursor of the charge transfer atoms-in-bonding expression. The homo- and heteronuclear atoms-in-bonding cases follow naturally as a generalization of PP former expression, while the conceptual–analytical advantage of the present approach is revealed in the homonuclear bonding case by surpassing the limitative zero charge transfer and energy in such bonding and elegantly avoiding the un-physicochemical picture of recording the redundancy of electronegativity and chemical hardness at both atomic and molecular levels;
- Section 3 deals with the application of the present atoms-in-bonding formalism for the halogen acids series of hetero- and homonuclear diatomic systems and the produced results are analyzed with the available experimental data both numerically and graphically. Finally, the tricky problem of molecular fragmentation and its reverse problem like the establishing of molecular pattern formation by its fragments and atomic groups is analyzed using the successive ad-atom methodology. To it we added the maximum chemical hardness jointly with minimum chemical power hierarchy to establish the most probable molecular channel formation by recursive atoms-in-bondings in polyatomic molecules, which were chosen as the prototype chemical ones (H_2_O, NH_3_, CH_4_, and C_6_H_6_).

Through the entire work, whenever possible, the connection with the actual or relevant trends in assessing atoms in bonding, atoms in molecule and their experimental fragmentation or pattern reconstruction techniques are appropriately documented for ensuring the enlarged base of addressability for the present endeavor—this way opening the challenge of the present findings in the context of experimental nano-research perspectives.

## 2. Conceptual Method

### 2.1. Orthogonal Space of Chemical Reactivity: The Chemical Power Concept

In modern theoretical chemistry, it is commonly accepted that for an electronic system we have the two complementary reactivity tendencies:

- electronegativity driving the tendency of the system in achieving electrons (so manifesting the reverse “chemical potential”); and
- chemical hardness controlling the tendency to inhibit the bonding and atoms-in-molecule coordination, being related with the system’ s chemical stability (so manifesting the “chemical inertia/force”).

**Figure 1:**
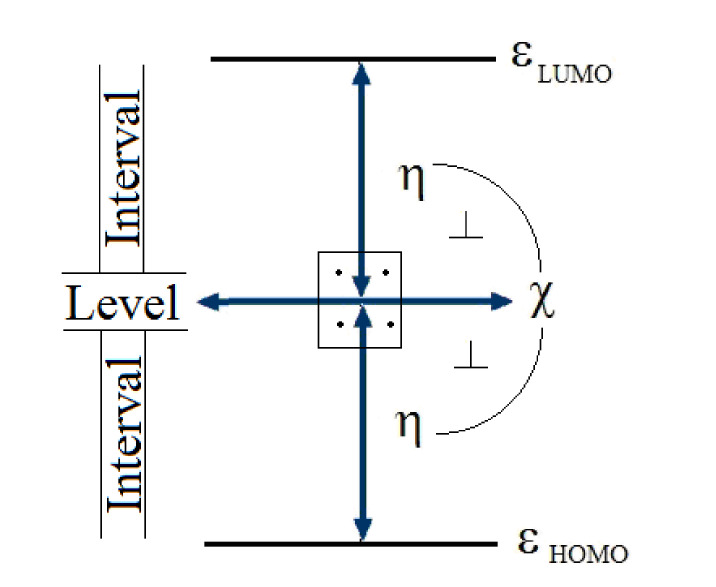
Electronegativity (χ) and chemical hardness (η) through orthogonal phenomena and related to HOMO-LUMO levels.

The two indices are energetically “orthogonal” (see Figure 1) because they are associated with a level and interval, in the adiabatic HOMO/LUMO spectroscopically modeling of Equations (1) and (2), respectively.

Having these tendencies in a “single dimensional space” usually creates a difficulty to decide what is the real (or the resultant) tendency for a given system (say *A*) characterized by its electronegativity ($\chi_{A}$) and chemical hardness ($\eta_{A}$) without combining them “in a higher dimension”. However, we need to consider a meaningful construct, so that one carefully inspects the general relationship between electronegativity and hardness, see the relation (2). To this aim we can introduce their nominal ratio, called chemical power ($C_{\pi }$), see references [45,46,54,55,56,57,58,59]:

(13) $$C_{\pi }=\frac{1}{2}\frac{\chi }{\eta }$$

At first sight, the chemical power relates with the maximum exchanged number of electrons, as earlier identified by Parr et al. while introducing the electrophilicity index [6], yet further insight is to be here revealed. The definition (13) gives us a sort of “reduced” or “normalized” electronegativity meaning that the real electronegativity or chemical reactivity for certain electronic system manifests when also its inertial hardness counts. The result provides a new global index indicating the real “power” of the system for attracting electrons. This is the qualitative interpretation of the chemical power.

**Figure 2:**
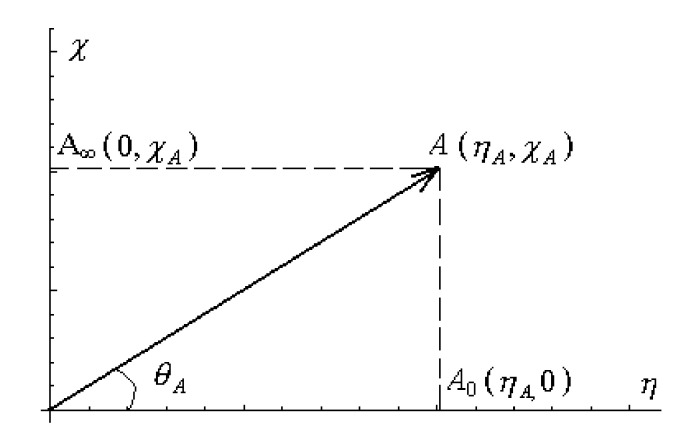
Cartesian representation of the η−χ (hardness-electronegativity) diagram for the electronic system *A* ($\eta_{A},\chi_{A}$) [45,46].

On the other hand, the quantitative meaning of the chemical power can be established by considering the Cartesian system with the coordinates being the hardness (on the abscissa) and electronegativity (on the ordinate). In this framework, the system *A* ($\eta_{A},\chi_{A}$) has the two projected points: *A*_0_ ($\eta_{A,}0$) on the hardness axis and A_∞_ ($0,\chi_{A}$) on the electronegativity axis, respectively (see Figure 2) [45,46].

Using Figure 2 and definition (13), the geometrical interpretation of the chemical power may take the following form:

(14) $$\begin{array}{ll} \tan(\theta_{A}) & =\frac{\chi_{A}-\chi_{A_{0}}}{\eta_{A}}\\ & =\frac{\chi_{A}-0}{\eta_{A}}\\ & =\frac{\chi_{A}}{\eta_{A}}-\frac{0}{\eta_{A}}\\ & =\frac{\chi_{A}}{\eta_{A}}\\ & =2C_{\pi }(A) \end{array}$$

From the equivalences of Equation (14) we arrive at the actual significance of the chemical power: the half of the total number of electrons that are shared by an electronic system *A* with electronegativity $\chi_{A}$ and chemical hardness $\eta_{A}$ with a system *A*_0_ that has the same hardness $\eta_{A}$ (so that the bonding is promoted by the celebrated hard-and-soft-acids-and-bases principle + by the tendency the electronegativity adducts’ difference be diminished in bonding) approaching the zero electronegativity. This way, the formal (yet insightful) definition of chemical power looks like

(15) $$C_{\pi }=\frac{1}{2}\left(\frac{\chi }{\eta }-\frac{0}{\eta }\right)$$

Equation (15) obviously recovers the former definition (13). However, with Formulation (15) rather than with the Equation (13), the evaluation of the chemical powers in the other “points”/states of the system *A* in Figure 2 produces the following respective results:

(16) $$C_{\pi }(A_{0})=\frac{1}{2}\left(\frac{0}{\eta }-\frac{0}{\eta }\right)=0$$

(17) $$C_{\pi }(A_{\infty })=\frac{1}{2}\left(\frac{\chi_{A}}{0}-\frac{0}{0}\right)=\infty -\frac{0}{0}$$

The chemical power result (16) in the point A_0_ appears as associated to the benchmarking system, i.e., the “electronic sea” of Parr et al. [6]. Instead, the result (17) it shifts to indefinite the divergent result for the chemical power in A_∞_ position, in the case it would be evaluated only on the Equation (13) basis. This way, the definition (15) gives the result (17) for the chemical point *A*_∞_ as far as the chemical hardness is absent in such a state. The conceptual consequence is important: no chemical system can be completely judged unless its chemical hardness is (including by zero value) specified, or in other words, the real chemical systems display quantifiable chemical inertia (force) represented by associated chemical hardness in all reactive or equilibrium circumstances. This is a result that shall clarify the current enmities regarding the viability in considering or not the chemical hardness among the working chemical descriptors in the favor of the affirmative.

We can thus conclude that for any given system *A* ($\eta_{A},\chi_{A}$) the chemical power $C_{\pi }(A)$ can be defined as the number of electrons that can be exchanged with the “chemically inertial” system *A*_0_ ($\eta_{A,}0$) having the same hardness value and zero electronegativity. It is thus a “referential” measure of the exchanged electrons in a chemical bonding and reactivity, thus affirming the “chemical power” in the earlier Parr and Yang spirit of “general chemistry” definition: modeling the displacement of electrons from one [substantial, referential] state to another [1].

The present approach introduces, within the 2D orthogonal space of electronegativity and chemical hardness the chemical action, Equation (13) as a dimensionless “super-potential,” so driving any envisaged reactivity; it gives an absolute figure to each chemical species (atoms, molecules, atoms in molecules) in focus and not (necessarily) relative to which interacts (unless electronegativity and chemical hardness of atoms-in-molecules or fragments-in-molecule are used, yet proven a larger degree of generality also this way). It may be therefore considered as another chemical-physical index on its own, as electronegativity and chemical hardness alone are—so it represents another physical basis from which the chemical reactivity picture may follow.

### 2.2. The Case of Heteronuclear Bonding (in Diatomic Molecules)

The first situation considers the diatomic molecule (or the chemical bond) *A–B* for which we like to determine the number of exchanged electrons, the average electronegativity, chemical hardness and the involved energy. The analysis develops on the above η−χ orthogonal framework.

**Figure 3:**
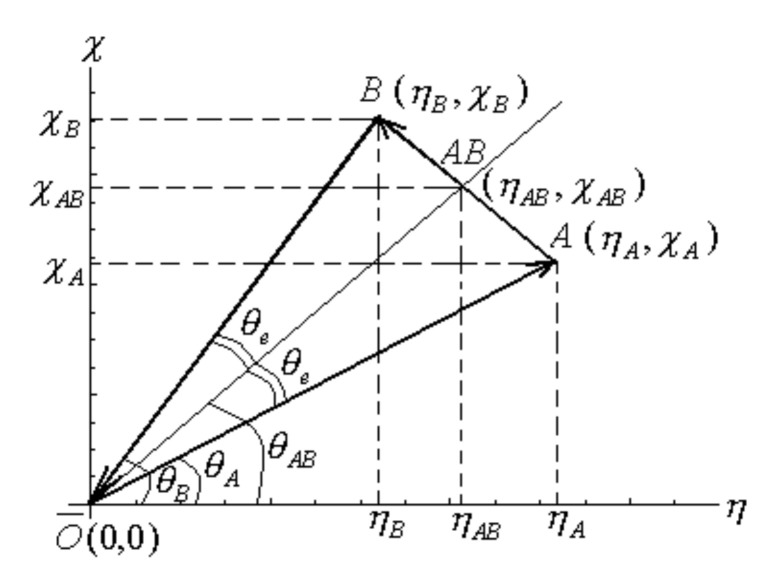
The chemical hardness-electronegativity (*η*-*χ*) orthogonal diagram describing the diatomic molecule/chemical bonding AB formation by charge transfer oriented flux.

Let us consider two atomic systems characterized by their hardness and electronegativity values through the points *A* ($\eta_{A},\chi_{A}$) and *B* ($\eta_{B},\chi_{B}$) in the η−χ Cartesian representation of Figure 3.

For determination of the electronic charge emerging by the A–B bond formation we chose a direction of charge flux in AB bonding in the allied η−χ diagram: it connects the reference point $\bar{O}(0,0)$, associated with the vacuum environment, with the points *A* ($\eta_{A},\chi_{A}$) and *B* ($\eta_{B},\chi_{B}$), as depicted in Figure 3.

The obtained closed contour can be interpreted as the “correlation order” of the constituents; in the present case, the phenomenological correlation order has the form: $\bar{O}\to )A\to B(\to \bar{O}$ from which appears that both atomic systems *A* and *B* have correlations with medium and between them too. The evolution in which these correlations are made is associated with the charge flowing direction when the *AB* bond is forming. Therefore, the *AB* charge transfer will be derived by the difference between the chemical powers of atoms *A* and *B* in the chosen correlation order, i.e.,

(18) $$\Delta N=C_{\pi }(B)-C_{\pi }(A)\;=\frac{1}{2}\left(\frac{\chi_{B}}{\eta_{B}}-\frac{\chi_{A}}{\eta_{A}}\right)\;=\frac{\chi_{B}\eta_{A}-\chi_{A}\eta_{B}}{2\eta_{A}\eta_{B}}$$

It is worth noting that $\eta_{A}\chi_{B}$ and $\eta_{B}\chi_{A}$ are not potential but squares of potential, and square of potential goes like couplings (scatterings) of potentials, i.e., $\langle k_{A}|{\hat{H}}_{A+B}|k_{B}\rangle ≅V_{A}^{-}V_{B}^{+}\sim \eta_{A}\chi_{B}$, when quantum-physically considering the (*A* + *B*) system Hamiltonian right-action on the ket-vector of wave-vector of the (valence) state of an adduct creating a particle/electron (thus favoring the electronegativity action), ${\hat{H}}_{A+B}|k_{B}\rangle ≅V_{B}^{+}\sim \chi_{B}$, and the left-action of the Hamiltonian on the bra-vector of the (valence) state of another adduct annihilating a particle/electron (so favoring the chemical hardness, chemical inertia and reactivity), $\langle k_{A}|{\hat{H}}_{A+B}≅V_{A}^{-}\sim \eta_{A}$. The present interpretation may be eventually related with quantum scattering matrix formalism of quantum many-body theory, a connection not to be here developed, yet in the pursuit of the future author’s research in quantum theory of chemical reactivity. However, by the mixed differences of the scattering products, namely, $\langle k_{A}|{\hat{H}}_{A+B}|k_{B}\rangle -\langle k_{B}|{\hat{H}}_{A+B}|k_{A}\rangle ≅V_{A}^{-}V_{B}^{+}-V_{B}^{-}V_{A}^{+}\sim \eta_{A}\chi_{B}-\eta_{B}\chi_{A}$, one recovers the numerator of Equation (18), yet with the more complex (quantum) information inside, giving the chemical charge transfer the quantum mechanical modeling of the quantum scatterings of the mixed influences that electronegativity and chemical hardness have among adducts to each other relatively to the global energy (Hamiltonian) of the adducts (atoms) in reactivity (molecule), see Appendix A.1.

On the other side, the charge transfer of (18) fixes the present figure of merit for the atomic correlation into the final average electronegativity and chemical hardness for the *AB* compound. In terms of COS (η−χ) diagram displayed in Figure 3, the resultant bond/molecule *AB* will have a chemical power fixed by the angle $\theta_{AB}$ that equilibrates the former angles $\theta_{A},\;\;\theta_{B}$, respectively. We chose here that such equilibrium takes place by arithmetic average formulation so maintaining the general framework in which the above Parr–Pearson bonding modeling was also constructed:

(19) $$\begin{array}{ll} \theta_{AB} & =\frac{\theta_{A}+\theta_{B}}{2}\\ & =\frac{1}{2}\left[\arctan\left(\frac{\chi_{A}}{\eta_{A}}\right)+\arctan\left(\frac{\chi_{B}}{\eta_{B}}\right)\right] \end{array}$$

With Equation (19), the first connection between the average electronegativity $\bar{\chi }$ and hardness $\bar{\eta }$ is then immediately

(20) $$\begin{array}{ll} \bar{\chi } & =\bar{\eta }\tan\theta_{AB}\\ & =\bar{\eta }\tan\left\{\frac{1}{2}\left[\arctan\left(\frac{\chi_{A}}{\eta_{A}}\right)+\arctan\left(\frac{\chi_{B}}{\eta_{B}}\right)\right]\right\} \end{array}$$

However, from Figure 3, there is clear that “the segment [*AB*]” has to contain the equilibrium point ($\bar{\eta },\bar{\chi }$); therefore, the second equation correlates the average electronegativity $\bar{\chi }$ and the average chemical hardness $\bar{\eta }$ is the equation of the [*AB*], by the analytical geometrical form

(21) $$\left(\bar{\chi }-\chi_{A})(\eta_{B}-\eta_{A})=(\bar{\eta }-\eta_{A})(\chi_{B}-\chi_{A}\right)$$

Now, the solution coordinates ($\eta_{AB},\chi_{AB}$) for the average chemical hardness $\bar{\eta }$ and electronegativity $\bar{\chi }$ are found at the intersection point between Equations (20) and (21), i.e., when Equations (20) and (21) express the same chemical reality. Observe that this procedure corresponds with the equalization principle in electronegativity in the Parr–Pearson picture. It also has the advantage that it furnishes at once both electronegativity and hardness values equilibrated in *AB* bonding/molecule, i.e., without assuming an additional “universality” correlation factors, as previously done by Equation (11).

Solving the system formed by Equations (20) and (21), we arrive at the following formulae for average electronegativity and hardness:

(22) $$\bar{\eta }\equiv \eta_{AB}=\frac{\chi_{A}(\eta_{B}-\eta_{A})-\eta_{A}(\chi_{B}-\chi_{A})}{\left(\eta_{B}-\eta_{A})\tan\left\{\frac{1}{2}\left[\arctan\left(\frac{\chi_{A}}{\eta_{A}}\right)+\arctan\left(\frac{\chi_{B}}{\eta_{B}}\right)\right]\right\}-(\chi_{B}-\chi_{A}\right)}$$

(23) $$\bar{\chi }\equiv \chi_{AB}=\eta_{AB}\tan\left\{\frac{1}{2}\left[\arctan\left(\frac{\chi_{A}}{\eta_{A}}\right)+\arctan\left(\frac{\chi_{B}}{\eta_{B}}\right)\right]\right\}$$

The remaining quantity to be determined is the transfer energy that accompanies the charge transfer (18) in forming the bonding/molecule *AB* with electronegativity $\chi_{AB}$ and chemical hardness $\eta_{AB}$. To this aim, we employ the fact that the electronegativity carries the negative energy in the first order of charge exchange ΔN, whereas the chemical hardness contributes parabolic by the charge exchange $\Delta N^{2}$ to the involved energy as recently conceptually and computationally validated [44,47]:

(24) $$\Delta E=\frac{1}{2}\left[-\chi_{AB}\Delta N+\eta_{AB}\Delta N^{2}\right]$$

Equations (18) and (22)–(24) are the present findings ones that correspond to Equations (8), (10), (12), and (9) in the Parr–Pearson approach, respectively.

### 2.3. The Case of Homonuclear Bonding (in Diatomic Molecules)

The next case is that one in which we deal with diatomic molecules in which the involved atoms are of the same type. Both Parr–Pearson and the present COS models are checked for the limits $\eta_{B}\to \eta_{A}$, $\chi_{B}\to \chi_{A}$. Accordingly, for the above Parr–Pearson approach, these limits give for the considered Equations (8), (10), (12), and (9) the following respective results:

(25a) $$\underset{\begin{array}{l} \eta_{B}\to \eta_{A}\\ \chi_{B}\to \chi_{A} \end{array}}{\lim}\Delta N^{\mathrm{Parr}–\mathrm{Pearson}}=0$$

(25b) $$\underset{\begin{array}{l} \eta_{B}\to \eta_{A}\\ \chi_{B}\to \chi_{A} \end{array}}{\lim}\eta_{AB}{}^{\mathrm{Parr}–\mathrm{Pearson}}=\eta_{A}$$

(25c) $$\underset{\begin{array}{l} \eta_{B}\to \eta_{A}\\ \chi_{B}\to \chi_{A} \end{array}}{\lim}\chi_{AB}{}^{\mathrm{Parr}–\mathrm{Pearson}}=\chi_{A}$$

(25d) $$\underset{\begin{array}{l} \eta_{B}\to \eta_{A}\\ \chi_{B}\to \chi_{A} \end{array}}{\lim}\Delta E^{\mathrm{Parr}–\mathrm{Pearson}}=0$$

Instead, for the present COS model, the corresponding Equations (18), (22)–(24) yield, respectively,

(26a) $$\underset{\begin{array}{l} \eta_{B}\to \eta_{A}\\ \chi_{B}\to \chi_{A} \end{array}}{\lim}\Delta N^{\mathrm{COS}}=0$$

(26b) $$\underset{\begin{array}{l} \eta_{B}\to \eta_{A}\\ \chi_{B}\to \chi_{A} \end{array}}{\lim}\eta_{AB}{}^{\mathrm{COS}}=\frac{\underset{\begin{array}{l} \eta_{B}\to \eta_{A}\\ \chi_{B}\to \chi_{A} \end{array}}{\lim}\left[\chi_{A}(\eta_{B}-\eta_{A})-\eta_{A}(\chi_{B}-\chi_{A})\right]}{\underset{\begin{array}{l} \eta_{B}\to \eta_{A}\\ \chi_{B}\to \chi_{A} \end{array}}{\lim}(\eta_{B}-\eta_{A})\underset{\begin{array}{l} \eta_{B}\to \eta_{A}\\ \chi_{B}\to \chi_{A} \end{array}}{\lim}\langle \tan\left\{\frac{1}{2}\left[\arctan\left(\frac{\chi_{A}}{\eta_{A}}\right)+\arctan\left(\frac{\chi_{B}}{\eta_{B}}\right)\right]\right\}\rangle -\underset{\begin{array}{l} \eta_{B}\to \eta_{A}\\ \chi_{B}\to \chi_{A} \end{array}}{\lim}(\chi_{B}-\chi_{A})}=\frac{0}{0}$$

(26c) $$\underset{\begin{array}{l} \eta_{B}\to \eta_{A}\\ \chi_{B}\to \chi_{A} \end{array}}{\lim}\chi_{AB}{}^{\mathrm{COS}}=\chi_{A}$$

(26d) $$\underset{\begin{array}{l} \eta_{B}\to \eta_{A}\\ \chi_{B}\to \chi_{A} \end{array}}{\lim}\Delta E^{\mathrm{COS}}=0$$

**Figure 4:**
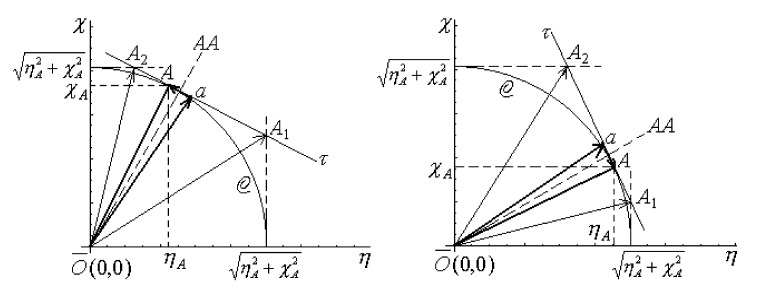
The same diagram as of Figure 3, yet here for treating mononuclear atoms *A_1_* and *A_2_* in covalent/molecular bonding *A-A*; one notices the circular curve (C) on which both atoms-in-bonding evolve; the stabilization of the chemical reactivity is by means of the diagram-left contributing atom for the left closing contour, or to the diagram-right for the right closing contour, respectively.

By one-to-one comparison, no essential differences between relations (25a)–(25d) and (26a)–(26d) are recorded, except for the average chemical hardness. For chemical hardness the indetermination is obtained in Equation (26b), i.e., confirming the same drawback for COS as for the Parr–Pearson model in the homo-atomic limit if taken just as mutatis-mutandis also for hetero-nuclear bonding. Fortunately, the present COS model allows the separate treatment for homo-atomic molecules by the key η−χ orthogonal diagrammatic representation. The related considerations start with the homonuclear geometric representation as in the COS of Figure 4.

In Figure 4, in the first step we evaluate the extreme coordinates for the chemical points’ *A*_1_ and *A*_2_.

- For the left-closing contour in Figure 4-left:
  (27a) $$\eta_{A_{2}}≅\eta_{A}-\frac{\chi_{A}}{\eta_{A}}\left(\sqrt{\eta_{A}^{2}+\chi_{A}^{2}}-\chi_{A}\right)$$
  (27b) $$\chi_{A_{2}}=\sqrt{\eta_{A}^{2}+\chi_{A}^{2}}$$
- For the right-closing contour in Figure 4-right:
  (27c) $$\eta_{A_{1}}=\sqrt{\eta_{A}^{2}+\chi_{A}^{2}}$$
  (27d) $$\chi_{A_{1}}≅\chi_{A}-\frac{\eta_{A}}{\chi_{A}}\left(\sqrt{\eta_{A}^{2}+\chi_{A}^{2}}-\eta_{A}\right)$$

Next, with Equations (27a)–(27d), one can form the analytical intersection between the geometrical segments [*A*_1_*A*_2_] and [*Oa*]; the intersection point is assumed close to the bonding equilibrium, or the pre-bonding point “*a*,” with the analytical coordinates

(28a) $$\eta_{a}=\frac{\eta_{A}^{2}+\chi_{A}^{2}}{\eta_{A}+\chi_{A}\tan\left[\frac{1}{2}\arctan\left(\frac{-\eta_{A}+\sqrt{\eta_{A}^{2}+\chi_{A}^{2}}}{\chi_{A}}\right)+\frac{1}{2}\arctan\left(\frac{\chi_{A}+\sqrt{\eta_{A}^{2}+\chi_{A}^{2}}}{\eta_{A}}\right)\right]}$$

(28b) $$\chi_{a}=\frac{\eta_{A}^{2}+\chi_{A}^{2}}{\chi_{A}+\eta_{A}\cot\left[\frac{1}{2}\arctan\left(\frac{-\eta_{A}+\sqrt{\eta_{A}^{2}+\chi_{A}^{2}}}{\chi_{A}}\right)+\frac{1}{2}\arctan\left(\frac{\chi_{A}+\sqrt{\eta_{A}^{2}+\chi_{A}^{2}}}{\eta_{A}}\right)\right]}$$

Finally, one treats the pre-bonding point “*a*,” with the coordinates of Equations (28a) and (28b), together with any of the two instantaneous contributing atoms in *A* ($\eta_{A},\chi_{A}$), in *AA* bonding, as in the final equilibrium bond by the model of Figure 3 and allied Equations, (18), (22) and (23); the final homo-nuclear bonding formulae are generated, respectively.

(29a) $$\Delta N_{AA}:=\Delta N_{aA}=\frac{1}{2}(\frac{\chi_{A}}{\eta_{A}}-\frac{\chi_{a}}{\eta_{a}})\;=\frac{\chi_{A}}{2\eta_{A}}-\frac{1}{2}\tan\left\{\frac{1}{2}\left[\arctan\left(\frac{-\eta_{A}+\sqrt{\eta_{A}^{2}+\chi_{A}^{2}}}{\chi_{A}}\right)+\arctan\left(\frac{\chi_{A}+\sqrt{\eta_{A}^{2}+\chi_{A}^{2}}}{\eta_{A}}\right)\right]\right\}$$

(29b) $$\eta_{AA}:=\eta_{aA}=\frac{\chi_{a}(\eta_{A}-\eta_{a})-\eta_{a}(\chi_{A}-\chi_{a})}{\left(\eta_{A}-\eta_{a})\tan\left\{\frac{1}{2}\left[\arctan\left(\frac{\chi_{a}}{\eta_{a}}\right)+\arctan\left(\frac{\chi_{A}}{\eta_{A}}\right)\right]\right\}-(\chi_{A}-\chi_{a}\right)}\;=\frac{\eta_{A}^{2}+\chi_{A}^{2}}{\eta_{A}+\chi_{A}\tan\left[\frac{1}{2}\arctan\left(\frac{\chi_{A}}{\eta_{A}}\right)+\frac{1}{4}\arctan\left(\frac{-\eta_{A}+\sqrt{\eta_{A}^{2}+\chi_{A}^{2}}}{\chi_{A}}\right)+\frac{1}{4}\arctan\left(\frac{\chi_{A}+\sqrt{\eta_{A}^{2}+\chi_{A}^{2}}}{\eta_{A}}\right)\right]}$$

(29c) $$\chi_{AA}:=\chi_{aA}=\eta_{aA}\tan\left\{\frac{1}{2}\left[\arctan\left(\frac{\chi_{a}}{\eta_{a}}\right)+\arctan\left(\frac{\chi_{A}}{\eta_{A}}\right)\right]\right\}\;=\eta_{AA}\tan\left[\frac{1}{2}\arctan\left(\frac{\chi_{A}}{\eta_{A}}\right)+\frac{1}{4}\arctan\left(\frac{-\eta_{A}+\sqrt{\eta_{A}^{2}+\chi_{A}^{2}}}{\chi_{A}}\right)+\frac{1}{4}\arctan\left(\frac{\chi_{A}+\sqrt{\eta_{A}^{2}+\chi_{A}^{2}}}{\eta_{A}}\right)\right]$$

The associated exchanged energy (24) in forming *AA* also becomes by the aggregate information of Equations (29a)–(29c):

(30) $$\Delta E_{AA}=\frac{1}{2}\left(-\chi_{AA}\Delta N_{AA}+\eta_{AA}\Delta N_{AA}^{2}\right)$$

Remarkably, now there is easy to check that when interchanging the coordinates of electronegativity and chemical hardness of Figure 4:

(31a) $$\underset{\begin{array}{l} \chi_{A}\to \eta_{A}\\ \eta_{A}\to \chi_{A} \end{array}}{\lim}\Delta N_{AA}=0$$

(31b) $$\underset{\chi_{A}\to \eta_{A}}{\lim}\eta_{AA}=\eta_{A}$$

(31c) $$\underset{\eta_{A}\to \chi_{A}}{\lim}\chi_{AA}=\chi_{A}$$

(31d) $$\underset{\begin{array}{l} \chi_{A}\to \eta_{A}\\ \eta_{A}\to \chi_{A} \end{array}}{\lim}\Delta E_{AA}=0$$

The previous Parr–Pearson homonuclear approach is exactly recovered. This further confirms PP theory as mono-dimensional (i.e., uni-parameter) dependency and in fact as a limiting case of the present 2D-COS approach.

### 2.4. Parr–Pearson vs. COS Approaches

It would be worth resuming the Parr–Pearson vs. present COS results, through side-by-side comparison, in the context of shortly reviewing the recent and currently available knowledge of chemical reactivity in terms of electronegativity and chemical hardness to a larger breadth:

- There is a general rule that larger molecules are chemically less harder (or softer) along congeners, when electronegativity is reduced or enhanced depending the substituent type; for example *χ* is decreasing by metal and mercapt on one side, and increased by phenyl, respectively, with direct consequence on physicochemical properties as the solvation free energy, or absorptivity on a surface, corrosion, etc., for nanomaterials [60];
- The reaction mechanism of functional groups, especially in organic chemistry, may be considerably understood when considering the electronegativity relationship with charge transfer and energy of atoms-in-molecules; see, for instance, the group electronegativity as well as the derived equations from the Sanderson equalization principles eventually applied to ionic groups [61];
- Chemical hardness provides a fruitful route for developing the density functional softness theory with the allied hierarchy for kernel, local, and global (including spin) reactivity indices, eventually leaving with new formulation of the linear response functions so useful in modeling the reactivity patterns of (open shell) molecules [62];
- The fashioned expressions of electronegativity and chemical hardness in their spectroscopic forms, i.e., 0.5 (I + A) and 0.5 (I − A), respectively, relay on the second order cutoff of the atomic energy Taylor expansion when ionization energy obeys the dependency I_n_ = nI_1_, with a wide applications on the A-Groups II, IV–VIII, excepting the oxygen [63];
- The electronegativity as the complementary chemical hardness influence determines also the super-molecule model of solute–solvent interaction and charge transfer, within the continuum polarizable charge model; actually, as the electrostatic potential diminishes by increasing of the effective radii of neutral and charged solute, the electronegativity equalizes between the solute and solvent and the chemical hardness diminishes too, so enhancing the chemical reactivity induced by solvent effects [64];
- Even the unconventional exohedral fullerenes as C_64_X_4_ (X = H, F, Cl, Br, and I) are subjected to chemical reactivity analysis through the basic electronegativity and chemical hardness indices: they both decrease with increasing atomic number of X in C_64_X_4_ cluster molecules. Instead, the CX fragment has electronegativity dependent on its located site. However, the electrophilicity index (ω = *χ*^2^/2*η*) increases from C_64_F_4_ to C_64_I_4_ paralleling the decreasing stability recorded by means of decreasing of bond dissociation energies, energy gaps, and maximal frequencies. They indicate a general nonaromatic character for the carbon halogen molecules of C_64_X_4_ (X = F, Cl, Br and I) [65];
- Electronic properties of oxides were successfully established in terms of the electronegativity- chemical hardness binomial (*χ*, *η*) coupling namely as (i) (*χ*↑ & *η*↓) is specific for oxides of transition elements with high oxidation state; (ii) (*χ*↑ & *η*↑) characterizes the insulators with large optical (HOMO-LUMO) gap; (iii) (*χ*↓ & *η*~>) feature alkali and alkaline earth oxides; and (iv) (*χ*↓ & *η*↑) describe ionic oxides with relatively large optical gap [66];
- The atoms-in-molecule Bent rule, according to which “more electronegative substituents prefer hybrid orbitals having less ‘s’ character and more electropositive substituents prefer hybrid orbitals having more ‘s’ character” was found consistent with the maximum chemical hardness reactivity principle, for a series of isomers, since the more electronegative atom occupies the axial position has maximum hardness. However, the same rule is less correlating with the minimum polarizability principle, being the last more sensitive to the basis set used, especially when the diffusion is this way better represented; application on different isomers of SF_4_, SF_4_O as well as for a family of PCl_x_F_5−x_ (x = 1–4) in combination with B3LYP with different basis sets of computational density functional theory confirms such general tendencies [67];
- On the other side, the simple MP2 model combined with the straight approximation of chemical hardness kernel as the Dirac localized function, $\eta (r,r^{\prime })≅\delta (r-r^{\prime })$, finely orders the Lewis acids and bases the computed global chemical hardness perspective when compared with experimental data [68].

In this context of chemical reactivity modeling by the electronegativity and chemical hardness coupling, it would be worth collecting the actual main results in both Parr–Pearson and COS pictures of atoms in bonding (diatomic molecules) by a common list of formulae (see Table 1).

One may start commenting on the side-by-side results of Table 1 from the Parr–Pearson vs. COS charge transfer interpretation, beside those given above in Section 2.2. For instance, one may notice that the induction and dispersion effects in chemical interactions both relate and root into the polarizability of chemical species in focus. However, the polarizability directly relates with electronegativity: low electronegativity goes with a very polarizable system, while high electronegativity associates with a not very polarizable system; on the other side, polarizability also inversely relates with chemical hardness, i.e., soft species (highly polarizable) correlate with low electronegativity, and hard species (not much polarizable) pose high electronegativity. Bearing this in mind, the actual approach uses the polarizability effects (and therefore including the induction and dispersion effects too) by the present geometrical denominator of Equation (18), $\Delta N_{AB}^{\mathrm{COS}}$ of Table 1, by product of adducts’ chemical hardnesses—consistent with the present scattering phenomenology by product of potentials in numerator (see Section 2.2). In other terms, one can say the actual mixing scattering potential difference (at numerator) is modulated by the resulted mixing polarizabilities (the inverse of the denominator chemical hardness product) in providing the charge transfer of Equation (18), $\Delta N_{AB}^{\mathrm{COS}}$ of Table 1, which further ignites the chemical reactivity. Instead, with the Parr–Pearson basic charge transfer of Equation (8), $\Delta N_{AB}^{\mathrm{Parr}–\mathrm{Pearson}}$ of Table 1, one is restrained only to addition of the adducts’ chemical hardnesses and therefore the resulting charge transfer appears modulated only by polarizability of the additive (superimposed) chemical hardness (chemical inertia) of the interacting systems, and not by mixing of their influences (more closely modeling the interaction phenomenology by the physical coupling of the potentials they represent).

Going further, the Parr–Pearson case arises as a limit from the present chemical orthogonal space relationships of Table 1:

(32a) $$\underset{B\to A}{\lim}\left({\mathrm{COS}}_{AB}\right)={\left(\mathrm{Parr}–\mathrm{Pearson}\right)}_{AA}$$

(32b) $$\underset{\begin{array}{l} \chi_{A}\to \eta_{A}\\ \eta_{A}\to \chi_{A} \end{array}}{\lim}\left({\mathrm{COS}}_{AA}\right)={\left(\mathrm{Parr}–\mathrm{Pearson}\right)}_{AA}$$

**Figure 5:**
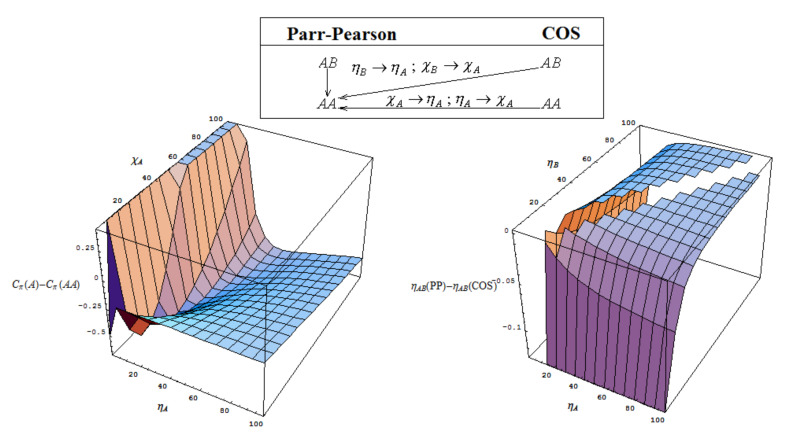
Top: synopsis of the COS to Parr–Pearson reduction; bottom-left: the chemical power difference of the isolated atom vs. atom-in-homonuclear bonding contribution; bottom-right: and the Parr–Pearson to COS chemical hardness difference for the *AB* bonding/molecule; all representations are made for equal unitary electronegativities $\chi_{A}=\chi_{B}=1$, i.e., modeling the bonding upon electronegativity equalization consumption, upon the formulas from Table 1 and chemical power Equation (13), respectively; see text for details.

Equations (32a) and (32b) can be interpreted as follows:

- The *AB* bonding case of COS consistently recovers the molecular *AA* case of Parr–Pearson;
- At the *AA* bonding level, the COS and PP approaches become identical when the chemical hardness and electronegativity interchanges their role by Equation (32b); this is in agreement with the reduction of the Figure 4 to Figure 3, so consecrating the present orthogonal chemical reactivity approach with the internal consistency (see also the forthcoming numerical applications in Section 3);
- Nevertheless, further interesting information may be extracted from the Table 1, especially on chemical power formulation, by Equation (13), resulting in Figure 5 with the variational meaning.

The chemical power from atomic (*A*) to bonding (*AA*) levels is graphically represented as $C_{\pi }(A)-C_{\pi }(AA)$ in Figure 4; its shape depends on input electronegativity ($\chi_{A}$) and chemical hardness ($\eta_{A}$) values; it clearly shows that the general range does not surpass 0.5 of charge transfer difference in passing from atoms-to-bond, a behavior in close agreement with the early findings [69]; yet it also correctly indicates the general propensity to the chemical bonding nature of atoms, by the ubiquitous $C_{\pi }(AA)>C_{\pi }(A)$ hierarchy; it also parallels the increasing of chemical hardness—that obviously stabilizes the atoms-in-bonding system and more on the COS side; this stands as another conceptual advantage of the present approach since there is better agreeing with the maximum chemical hardness principle;

Thorough the $\eta_{AB}(PP)-\eta_{AB}(COS)$ representation of Figure 5 also the “*AA* hole” in PP treatment appears graphically identified (see Equation (26b)). Overall, the COS approach provides superior chemical hardness and chemical reactivity information respecting the PP framework.

Moreover, it is worth noting that the same mixing potentials (couples) $\eta_{A}\chi_{B}$ and $\eta_{B}\chi_{A}$ appear also in the Parr–Pearson average electronegativity in Equation (10), $\chi_{AB}^{\mathrm{Parr}–\mathrm{Pearson}}$ of Table 1—yet as a sum, as a consequence, while in our Equation (18), $\Delta N_{AB}^{COS}$ of Table 1, they stay as a driving coupling potential: their mixed difference is the driving “scattering potential”, see below, more complex than the potential difference alone of Equation (10), $\chi_{AB}^{\mathrm{Parr}–\mathrm{Pearson}}$ of Table 1. Therefore the present difference of the mixed scattering potentials (see also Appendix A.1) $\eta_{A}\chi_{B}$ and $\eta_{B}\chi_{A}$ appearing in $\Delta N_{AB}^{COS}$ of Table 1 are placed as *the cause* of the reactivity—not as an effect of it—as appearing in the Parr–Pearson approach by the resulting (as an effect) average in electronegativity of Equation (10), $\chi_{AB}^{\mathrm{Parr}–\mathrm{Pearson}}$ of Table 1. Accordingly, the present approach is a changing in paradigm of chemical reactivity, rooting in quantum mechanics (see above), yet with all dimensionality and analytics correctly developed.

More applied discussion will be given just below with the occasion of numerical realization of PP vs. COS chemical bonding characterization of given molecular systems and of their fragmentation patterns.

## 3. Results and Discussions

Before going on to concrete numerical applications of the current COS vs. PP electronegativity and chemical hardness chemical bonding indices, it is worth reviewing the recent advances in modeling chemical bonding either by structural or reactivity indices. This way, we may also quote some selected experimental approaches regarding atoms or fragments in bonding. It also offers the prequel for the fragmentation identification techniques and reciprocal molecular patterning while creating the physical-chemistry conceptual and computational environment in which the present applications can be placed with possible further connections and multi-scaled approaches by nano-chemistry.

- The standard B3LYP/6-311G++(d,p) method along the basis set calculation within density functional theory for reactivity indices as ionization potential (IP), electron affinity (EA), electronegativity (*χ*), chemical hardness (*η*), and the electrophilicity index (ω) have proved to correlate with HOMO and LUMO energies. They relate the molecular spectroscopic properties such as vibrational spectral analysis, e.g., carried out by Raman and infrared spectroscopy in the range 4000–400 cm^−1^ and 3500–100 cm^−1^, respectively, for the 2-hydroxy-5-bromobenzaldehyde (HBB), a benzaldehide derivative with potential action in anti-microbial activity ant-cancer properties, to reveal that the charge transfer mainly occurs within the molecule, as based on the produced electrostatic potential maps [70];
- Natural bond orbital analysis (NBO, NBA) may be carried out for establishing the charge transfer between the localized bonds and lone pairs; their calculated electronic and optical properties, i.e., absorption wavelengths, excitation energy, dipole moment, molecular electrostatic potential (MEP), and frontier molecular orbital energies correlate with the observed X-ray diffraction, FT-IR, and UV-Vis spectroscopically methods; as a result, the elucidation of the intramolecular hydrogen bonded interactions may be established, for instance, strong O–H···N interactions in enol-imine form and N–H···O interactions in keto-amine for Schiff base compounds (such as (E)-2-((4-hydroxy-2-methylphenylimino)methyl)-3-methoxyphenol) exhibit unique properties in catalysis and medicinal chemistry [71];
- Schiff bases generally display biological activity superior to the free ligands, with direct application to the study of DNA binding, cytotoxicity and apoptosis. Therefore, they become viable alternative for common chemosensors used in molecular recognition and anion sensing; this is due to the manifested tautomerism of the OH group in ortho position as well the imino group in both solution and solid states, especially at the level of the hydrogen bonds O-H···N or O···H-N between the enol-imine and keto-amine forms, respectively [72];
- Charge reversal of cations (^+^CR^−^) stands as a complementary method in the arsenal of mass spectroscopy methods used in distinguishing the prototype hydrocarbon cations, such as C_6_H_5_^+^ and C_6_H_6_^+●^; the last compound is probably the most studied ion by coincidence measurements, collision- and surface-induced dissociation, charge-stripping, neutralization–reionization, and ion–molecule reactions spectrometric methods [73];
- The charge transfer may be considered to be the driving parameter for the adatom–metal bond strength, thus anticipating the adsorption energy trends in terms of electronegativity and chemical hardness. The work function is used for the metal surface electronegativity, with the electronegativity of metal being a combination of the atomic Mulliken electronegativity and of the work function of the metal surface. The resulting charge transfer is viewed as the metal surface and adatom electronegativity difference These results further consecrate the HSAB principle in terms of charge transfer parameter *Δ*N [74].

### 3.1. Diatomic Molecules with Hetero- and Homo-Atoms

In order to have a proper numerical comparison between the actual chemical orthogonal space and the fashioned Parr–Pearson atoms-in-bonding picture, we first consider the diatomic case by analyzing the halogen acids series: HF, HCl, HBr, HI, ClF, BrF, IF, BrCl, ICl, IBr. Accordingly, the application to the formulae of Table 1 and of the chemical power figure of merit with Equation (18); are displayed in Table 2. In Table 2 we notice the close values between the values of average hardness and electronegativity in both Parr–Pearson and present COS approaches, in all considered diatomic cases. However, the actual COS treatment gives simultaneously the expressions for molecular electronegativity and chemical hardness, without the additional constrains of Equation (11) in Parr–Pearson model.

However, for the charge transfer and exchanged energy pair (*Δ*N, *Δ*E) we note interesting discrepancy: while the Parr–Pearson model allows unphysical equal sign variation, i.e., (−,−), the COS treatment always gives the systematic of opposite sign in charge and energetic variations, (+,−) or (−,+), as it should physically be modelled, respectively. Moreover, the molecules HBr and HI display the same amount of exchanged charge (0.02) with opposite signs, whereas the related exchanged energies have the same sign and stabilization value (−0.004), respectively—a physically difficult to interpret instance. Instead, the present COS model correlates in a consistent manner the sign of the charge transfer with the opposite sign in exchanged energy, e.g., when the atoms *B* in above examples receive the transferred charge in bonding it manifests the affinity behavior, so with the negative sign for allied energy exchanged, corresponding with the ground state stabilization. The reverse situation holds for the released charges (negative sign for transferred charge, in Table 2, i.e., from the *B* atoms) that are accompanied by an uptake energy (triggering the *B* systems to lose charges) at the level of the valence state. Therefore, present COS model may enlarge the limitative Parr–Pearson approach and the enmities around it due employing a parabolic form of energy of ground state to what it would be more appropriate be considered as valence state (see refs. [5,6]). Nevertheless, this way, the standard enthalpy of formation (and the related thermodynamic quantities) may be further employed for correlation since both valence and ground states interplay for atoms-in-molecule takes place, so allowing activation states, reorganization of charge and switching among chemical bonding types, etc.—the matter remains open for further analysis.

In this context, there is quite evident that apart of the HF, HCl, and HBr molecules for all other cases in Table 2 the Parr–Pearson and the present COS model predict different directions of charge flowing in forming *AB* bondings/molecules. In order to explain this dichotomy, we notice that in the same class of compounds (the acidic halogens in Table 2) only HI is manifesting with the charge flux more oriented to the H atom in HI molecule within the Parr–Pearson model: this because the PP model is exclusively based on the electronegativity difference/equalization between the molecules’ components, and without chemical hardness evolution (i.e., as HSAB → maximum hardness).

On the other hand, it is clear that the chemical hardness controls the stability (starting from the atomic level) so that it is worthwhile to consider it as an influential coordination dependency. In this regard, the present COS approach, through the chemical power concept (see Equation (11)), describes the charge transfer within a framework in which both electronegativity and hardness information are complementarily (orthogonally) evolved.

**Figure 6:**
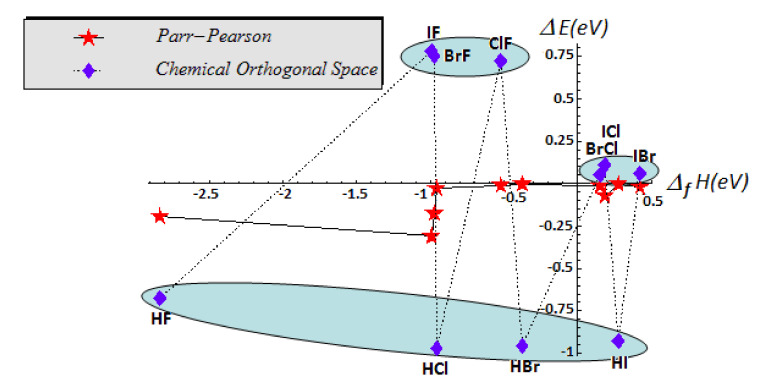
Estimated energies within Parr–Pearson (PP) and chemical orthogonal space (COS) vs. standard enthalpy of formation (*Δ_f_H*) at 298.15 K for the halogen acids of Table 2, manifesting better molecular ground-to-valence states’ sub-classification for the later, respectively.

The last remark about the numerical results reported in Table 2 regards the exchanged energy trend in the same group of halogen acids (HF, HCl, HBr, HI): being about the same class of combination, the fact that the exchanged energy varies (absolutely) from 0.21 for HF to 0.004 for HI is again difficult to interpret by PP approach; if truly so, it would mean that the HI and HBr molecules are formed almost without spending of energy from atoms, so that the atoms arranges in molecules without interacting in fact. Instead, the present COS model gives out, for a given class of compounds, the same scale of energetic exchange for bonding formation; this behavior is also illustrated by the joint graphical trends of energy respecting the gas phase standard enthalpy of formation (*Δ_f_H*) at 298.15 K for the data in Table 2 and reported in the Figure 6. This representation clearly illustrates the sub-class separation, i.e., (IF, FBr, FCl) in valence state vs. (HF, HCl, HBr, HI) in ground state within COS rather than within the PP framework.

**Figure 7:**
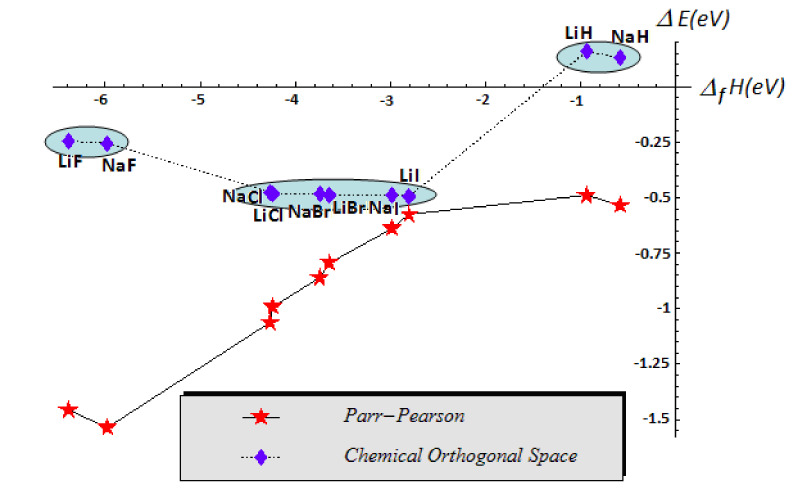
The same type of representations as in Figure 6, yet for alkaline hydrides and salts as polar molecules in a crystal state.

Similar analysis may be performed for the alkaline hydrides and salts of Table 3, with the energetic correlation reported in Figure 7. In this case, the COS approach shows the parallel evolution of the charge with energy exchange in bonding, yet with more similarity in sign and sub-class correlation respecting the PP evaluations; this is clear also from the Figure 7, where the same kind of molecules are identified in the same sub-class of ground-states, paralleling the standard enthalpy of formation (*Δ_f_H*). It is worth noting that the ionic hydrides of Figure 7 are distinct in COS approach featuring negative charge transfer value; this is the sign that they bears a certain degree of ionicity in resonance with covalency, i.e., $\mathrm{LiH}↔Li^{+}H^{-}$, $\mathrm{NaH}↔Na^{+}H^{-}$. A quantum-mechanical explanation may lie on negative hole function, at its turn in terms of the correlation probability the electronic pairing having a non-stable (i.e., fluctuating) localized functions [8] or, in simple terms, the adducts fragment/natural AOs did not have stable wave function.

**Figure 8:**
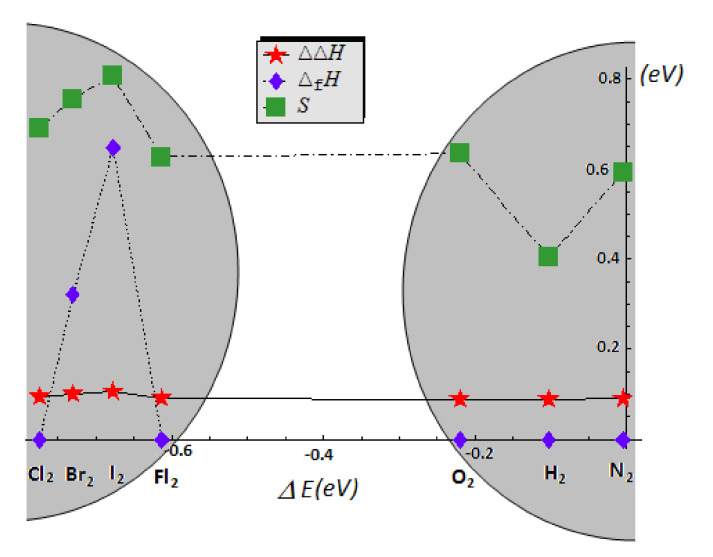
The same type of representations as in Figure 5 and Figure 6, yet for the homonuclear diatomics of Table 4. Only the COS values are provided since all PP values vanish according to Table 1 for homonuclear bonding.

Finally, the homonuclear diatomics are considered by the prototype molecules of Table 4, with the same chemical bond by chemical reactivity information as reported in Table 2 and Table 3. For experimental reference, the entropy is also added, while the standard enthalpy of formation (*Δ_f_H*)—displaying many zeros across the series, was supplemented with the referential enthalpy difference *ΔΔ*H = H(298.15 K) − H(0). The graphical representation of the experimental thermodynamic energetics vs. COS (non-zero) binding energies of Table 4 is given in Figure 8. The halogen diatomics appear as a distinct sub-group of molecules, widely separated from the second group gaseous covalent molecules in all thermodynamic frameworks. Obviously, the PP approach provides no information as such by its inherent limitation of (vanishing) values for homonuclear bonding.

### 3.2. Atomic Paths in Molecular Formation

Eventually, the present COS atoms-in-bonding paradigm may be further used in establishing the forming channels of poly-atomic molecules, beyond the diatomic cases in either hetero- or homonuclear combinations. At this point, the conceptual problem is the same as in the molecular fragmentation identification in experimental observations and equally of its inverse problem—namely, the molecular reconstruction with highest probability by the fragments’ inter-correlation patterns. Therefore, as before, it is worth noting the COS numerical applications on add-atomic and add-molecular fragments phenomenology with the representative findings in molecular fragmentation and pattern recognition by experimental (usually mass spectroscopy) techniques:

- The resolution mass spectroscopy investigates the mechanisms through which fragment ions are formed; in particular, the remote site fragmentation with charge localization can be useful for identifying ions containing portions of molecule, e.g., ammonia chemical ionization for cardiac glycosides [77];
- Fourier transform mass spectrometry may indicate the rapid adduct formation through extrusion reactions, e.g., the direct extrusion of CO from furan, or HCN extrusion from pyrrole, by the aid of hydrogen-atom displacement dominant reaction, eventually followed by additional neutral loses [78];
- Molecular fragmentation may be produced by ionic-molecular collision at keV projectile energies, while the range of eV corresponds to the Coulomb explosion of ionized target, e.g., (H_2_O)^q+^ in He^2+^ +H_2_O reaction, sometimes with smaller absolute cross section than the classical Rutherford formula, due to the screening of the bound electrons [79];
- Atoms-in-molecule properties, such as zero-point kinetic energy, may be determined by high-resolution electron Compton scattering combined with the Doppler broadening recording of the atomic momentum distribution in molecule contributing to both external and internal molecular motion, respectively; e.g., the e-scattering intensities from H- and O- atoms in molecular systems such as H_2_, CH_4_, H_2_O, and NH_3_ [80];
- Molecular fragments may also have an important role in selective catalytic reduction, eventually monitorized by the spectroscopic techniques such as an in situ FT-IR spectrophotometry; e.g., the case of NH_x_ species production by the introduction of H_2_ into the reduction of NO over Pt-MnO_x_ catalysts [81];
- The pressure effect on the molecular fragment dynamics over the catalyst is especially present in the final thermodynamically part of the reaction, approaching its equilibrium, and not in its first part when the kinetics is dominant by the mass transfer and consumption of the O_2_ and fuel; e.g., the case of catalytic partial oxidation of light hydrocarbons, e.g., the CH_4_ and C_3_H_8_, eventually leading with the intermediate species C^2+^ [82];
- Bifunctional molecules (e.g., CH_3_OCH_2_COCH_3_ and CH_3_COCOCH_3_) are in general found to be less stable in low temperature solids as compared with the simple generic compounds, by the so called “hot” fragmentation with a probability decreasing with the excess energy diminishment, down group of periodic system. This is the case of solid rare gas matrices, e.g., yielding ${}^{•}{CH}_{3}$ radicals upon the irradiation in solid Ar matrix at T < 16K [83];
- Preferential breaking of the strain bonds by laser ablation is an effective experimental method for controlling the molecular fragmentation, especially on good surface heating in the absence of any direct absorbance on the surface by from the concerned strained compound, e.g., ring compounds deposited on metal rods [84];
- Ligand elimination and ligand decomposition channels can also be approached by laser vaporization through laser photo-dissociation; even in exotic complexes such as bare uranium cations U^+^(C_6_H_6_)_n=1–3_ and uranium oxide ions UO_m_^+^(C_6_H_6_)_m=1,2_, the first one is more effective in expelling the neutral benzene in a ground state from the excited U^+^, U^+^(C_2_H_2_), and U^+^(C_4_H_2_) fragments’ decay, once they are formed under ultraviolet action on the former uranium complex [85];
- The main molecular dissociation channels may be established both by density functional calculation as well as from experimental electrospray mass spectroscopy fragmentation spectra; the consecrated example is that of protonated uracil (UH+) fragmentation using collision at increased energy, leading mainly to extraction of isocyanic acid (HNCO, 43Th) from the aromatic (cationic uracil) cycle, and yielding the C_3_H_3_NO fragment of 69-Th by means of the retro Diels-Alder reaction mechanism [86];
- Approaching the identification of the fragmentation mass spectra that are not contained in spectral library is under current focus in order to overcome the limits of the “known universe in organic chemistry” with huge consequence in assessing the genomics, proteomics, and metabolomics open issues. To this aim the extensive computational approach is replaced with similarity and fitting studies while having the molecular structure and allied reactivity properties such as reactivity indices of electronegativity and chemical hardness in their forefront [87].
- Conceptual-computational methods are truly effective in establishing the molecular inverse problem, i.e., determining the parent molecule (when searching for elucidation of structure of new/unknown materials) by simulated fragmentation pathways; for instance, the Simplified Molecular Input Line Entry Specification (SMILES) correlates satisfactorily with peak evolution in Gentle Secondary Ion Mass Spectrometry with fragmentation pathway mapping (G-SIMS-FPM) methods, as was essentially found for simulated pathways for amino acids and simple peptide [88]. A similar method was used also for studying the folic acid (with its six subunits: *α*, *β*, *γ*, *δ*, *ε*, *ζ*) and Irganox 1010 (a central carbon atom surrounded by four equal side-chains) through varying the G-SIM surface plasma temperature aiming producing new data-based system (including amino acids and simple peptides). The acquisition of fragmentation pathway simulation/molecular structure re-assemble information (about 90% of the fragments explained) plays a major role in analyzing the interaction in bio-molecule (protein) surface by means of polymer, drug-delivery, and organic electronics technologies [89];
- The synergy of atoms-contributing-catalysts as are Ni and Cu in NiO formation by plasma-treated sample of Ni-CU/Al_2_O_3_ nanocatalyst features enhanced reactivity compared with just impregnated nanocatalysts, since more uniform morphology recorded (e.g., by XRD and TEM techniques) for the first case, not withstanding that these nanomaterials lose about 10–12% of CH_4_ and CO_2_ through conversion, respectively, during the overall time of stream test due to the methane decomposition and decrease the RWGS (reverse water gas shift) reaction rate, while gas hourly space velocity (GHSV = Reactant Gas Flow Rate/Reactor Volume) has less effect on the reforming reactions for plasma-treated sample due to well-defined morphology of the nanocatalyst [90];
- Subtle aspects of energetics of intra- and inter- fragment density rearrangements, charge transfer and orbital mixing was recently observed through computational experiments of Wernerian complexes in the bonding regime of ligand field effects allowing new quantum insight in understanding the ligand field stabilization energy (LFSE); the method involves the energetic decomposition of the metal ion by ligand sets with fractional charges as resulted upon preliminary electronegativity equalization effect driven by charge transfer, thus producing their separate nominal oxidation states. This way, the 10Dq separation for prototype octahedral units (such as [M^q^F_6_]^q^^−6^, [M^q^(CN)_6_]^q^^−6^, and [M_q_(H_2_O)_6_]^q^ complexes with M^II^ and M^III^ ions selected from the M = Cr to Cu 3d series) was predicted by LFSE and electron promotion effects, leading to the interesting interplay between the ionic and covalent bonding regimes in coordination bonding characterization [91];
- The metal-benzene bonding clusters, i.e., Fe_4_–(C_6_H_6_)_m_, m ≤ 3, were characterized through metal (iron)–carbon bonding, driving the contour plots of molecular orbitals, while noting some isolated forbidden IR modes nearby those of isolated benzene; yet, IR activated ligand regime, as due to the 2p-π electrons of benzene interacting with the 3d electrons of Fe_4_ estimates ionization energies, electron affinities (so combined into electronegativity) and (related) binding energies in good agreement with experimental data [92];
- Fragmentation of carbon based molecules (such as glycine C_2_H_5_NO_2_) by slow (low-energy) monoenergetic electrons have been investigated with particular focus on the mechanism of formation of the doubly charged fragment (e.g., CH_2_NHCO^2+^); unusually, they are not detected by mass spectroscopy, since geometrical rearrangement of the initial molecule; however, they are accompanied by the C-H bond breaking with yield of the [OH + H] fragment [93]; the same mechanism and doubly charged fragment ionic formation was advanced also for methionine (C_5_H_11_NO_2_S) molecule with the only difference that the main channel of dissociation involves the sulfur atom, eventually leaving with the formation of the CH_4_S^+^, while the dominant channel formation of the C_2_H_5_S^+^ ionized fragment favors the charge stabilization of the sulfur atom [94];
- The mass analyzed threshold ionization (MATI) technique is another spectroscopic tool used for assessing the molecular fragmentation structure, especially for molecules with biological relevance by their manifested π-hydrogen bonded clusters, e.g., localization of the amino hydrogen in the pyrole ring of 3-methylindole·C_6_H_6_ cluster (58 018 cm^−1^) is comparable with the results obtained for 3MI·C_6_H_6_ cluster, thus confirming the methylation influence on the π-hydrogen bonding [95];
- The ultraviolet dispersed fluorescence spectroscopy was employed to provide the photo-fragmentation of H_2_O for photon excitations over 20eV; the identification of channel transition *A*^2^*Σ^+^*→*X*^2^*Π_Ω_* for the OH fragment at higher energy about 30eV is in good agreement with vertical Rydberg states of water, but overcoming the dissociation limit for the dissociation channel OH(*A*^2^*Σ^+^*) + H^*^(n ≥ 2) [96].

All these experimental techniques encourage the pursuit of modeling add-atoms in molecules in a recursive way, promoting add-fragments in molecular systems and bond formation; the special relevance would be in solid state chemistry, e.g., by surface add-atom interactions, and allied electronic transfer and conduction’s interactions; viz. the bilayer graphenes coupled by organic/dyes molecules, and further hybrid materials and hetero-junctions combinations with envisaged increasing photo-voltaic effects.

Accordingly, for actual COS implementation, one needs to follow three components of a given poly-atomic add-atom analysis:

(i). Given a poly-atomic molecule, or a complex chemical system, the various add-atomic and add-bonding recursive combinations are considered in various channels patterning the final target molecule;
(ii). Since the stabilization of the molecule is described by the maximum chemical hardness principle, the chemical hardness hierarchy is considered for the various channels in stage (i). It is nevertheless estimated through recursive pairs of add-atoms and add-bonds in the pursued patterns;
(iii). The maximum chemical hardness principle is further combined with chemical power information, for which the associated minimum variational principle shall be applied: as based on the Equation (13), the chemical power contains also the electronegativity information, and of its minimum variation around equilibrium too, for the same channels patterning as analyzed in previous steps (i) & (ii).

This procedure is here employed for the typical molecules H_2_O, NH_3_, CH_4_, and C_6_H_6_ since they were also the subject of the above-summarized experimental works on molecular fragmentation and patterning. The identified channels are characterized according with the step (i) above and numerically modeled along the reactivity information and chemical power indices, see Table 5, Table 6, Table 7 and Table 8, respectively. The steps (ii) and (iii) will be commented in the sequel, with the general note that the chemical power registers the custom PP ≥ COS hierarchy, so restraining the present analysis to the COS recursive framework as the best one for maximum chemical hardness principle realization.

For the water molecule, with the molecular add-atom and add-bonding patterning revealed in Table 5 identifies the maximum chemical hardness and minimum chemical power hierarchy, respectively as

(33a) $$\eta (2)>\eta (3)\;\mathrm{vs}.\;C_{\pi }(2)<C_{\pi }(3)$$

Equation (33a) points to the recursive add-atomic formation of the water molecule though the identified molecular inverse fragmentation path:

(33b) $$C_{\pi }(2):\;(H+O)+H\;\to \;H_{2}O$$

The add-atom and add-bonding channels of forming ammonia molecule are displayed in Table 6; from it, the abstracted variational hierarchies for chemical hardness and chemical power are accordingly found to be

(34a) $$\eta (5)>\eta (6)<\eta (4)\;\mathrm{vs}.\;C_{\pi }(5)<C_{\pi }(6)<C_{\pi }(4)$$

From Equation (34a), again, the anti-parallel evolution of the chemical hardness and chemical power identifies, from the recursive chemical bonding and reactivity perspective, the add-atomic channel with add-bonding (here as the hydrogen molecule) patterning of ammonia formation:

(34b) $$C_{\pi }(5):\;(N+H)+H_{2}\;\to \;{\mathrm{NH}}_{3}$$

The add-atoms and add-bondings channels of Table 7 modeling the methane patterning provides a slightly shift between the maximum chemical hardness hierarchy

(35a) η(11)>η(7)>η(9)>η(10)>η(8)

and the corresponding minimum chemical power ordering

(35b) $$C_{\pi }(7)<C_{\pi }(11)≅C_{\pi }(9)<C_{\pi }(8)<C_{\pi }(10)$$

In such a situation, the final cut is given by the chemical power hierarchy since it includes the maximum chemical hardness principle supplemented by the electronegativity information (and allied variational principle); in this case, the resulting $C_{\pi }(7)$ case appears as the overall minimum of chemical power among all patterning channels of methane formation of Table 7; it yields the model of double (recursive) add-atomic adducts with the add-bonding of the hydrogen molecule in the final stage (in the same manner as was previous the case with ammonia) targeting the methane formation

((C + H) + H) + H_2_ → CH_4_ (35c)

The final case illustrated in the present work is the benzene molecule. It is patterned through the channels of Table 8, from which, following the previous lesson regarding maximum of chemical hardness vs. minimum of chemical power hierarchy—only the last one is considered.

(36a) $$\begin{array}{l} C_{\pi }(6)<C_{\pi }(10)<C_{\pi }(11)<C_{\pi }(7)<C_{\pi }(9)<C_{\pi }(8)<C_{\pi }(2)\\ <C_{\pi }(5)<C_{\pi }(13)<C_{\pi }(12)<C_{\pi }(4)<C_{\pi }(14)<C_{\pi }(3)<C_{\pi }(1) \end{array}$$

This way, the patterning for the closing ring of benzene is readily selected through the add-bondings of acetylenes—eventually rearranged in an aromatic structure.

(36b) $$C_{\pi }(6):\;C_{2}H_{2}+C_{2}H_{2}+C_{2}H_{2}\;(+C_{2}H_{2})\;\to \;C_{6}H_{6}$$

The present results are conceptually and computationally reliable and consistent. Nevertheless, they should be considered under future scrutiny of experimental investigation and possible confirmation. If the present chemical orthogonal space will be proved as a chemical reality it will represent another leap in the chemical modeling not reducible to the physical (quantum) pictures yet preserves its algebraic (and analytical geometrical) formalism and value.

### 3.3. Open Issues

The present model may be seen as a conceptual compromise among the dependency and independency of the electronegativity and chemical hardness chemical reactivity indices, since “orthogonal averaging” over:

- Their interrelation through the basic definitions as variation respecting the charge exchange in a reactive/boning system;
- Their apparent disjoint measures of interpreting chemical reactivity and bonding completion, viz. the minimum variation principle of electronegativity, Δχ≤0, and the maximum variation principle of chemical hardness, Δη≥0 Refs. [1,2,3,35].

This is the key with which many of the open issues of chemical reactivity and bonding can be unfolded and further studied, among which some relevant examples are discussed in the next section.

#### 3.3.1. Computational Context for Generating Parameters

The present study employs as the input parameters the electronegativity and chemical hardness used by Parr and Pearson’s earlier developments [1,2,5]; they are, however, in their turn based on the first order finite approximation of the corresponding charge derivatives, Equations (1) and (2), respectively, i.e., on the first order ionization potential and chemical affinities, spectroscopically—so experimentally estimated, respectively. This approach can be considered as the first order approximation of the model and all its comparative unfold; it is actually quantum-mechanically based on the frozen core orbitals by the Koopmans Theorem [7,17]; yet, even in this context, more complex/compact schemes of finite difference (CFD) development may be considered, until the so-called spectral-like resolution expansions, there where up to the third orders of ionization potentials (HOMOs) and ionizations affinities (LUMOs) are involved [18,41]. Still even in such orders, the orthogonality relationships are preserved since the electronegativity and chemical hardness are exclusively under semi- sums and semi-differences, respectively,

(37a) $$\chi_{CFD}=f\left(\frac{\varepsilon_{HOMO(1)}+\varepsilon_{LUMO(1)}}{2},\frac{\varepsilon_{HOMO(2)}+\varepsilon_{LUMO(2)}}{4},\frac{\varepsilon_{HOMO(3)}+\varepsilon_{LUMO(3)}}{6}\right)$$

and

(37b) $$\eta_{CFD}=g\left(\frac{\varepsilon_{LUMO(1)}-\varepsilon_{HOMO(1)}}{2},\frac{\varepsilon_{LUMO(2)}-\varepsilon_{HOMO(2)}}{4},\frac{\varepsilon_{LUMO(3)}-\varepsilon_{HOMO(3)}}{6}\right)$$

note that the chemical orthogonality, as defined in the present report, is lost when the Koopmans approximation is abolished, and the spectral levels are allowed to relax during the ionization/affinity chemical reactivity and bonding processes. In such cases, for instance, the second order ionization potential and electron affinity become as in Ref. [3] (Volume 3):

(38a) $$IP_{2}=E_{N-2}-E_{N-1}=-\varepsilon_{HOMO(2)}+\langle HOMO_{1}\;HOMO_{2}|HOMO_{1}\;HOMO_{2}\rangle$$

(38b) $$EA_{2}=E_{N+1}-E_{N+2}=-\varepsilon_{LUMO(2)}+\langle LUMO_{1}\;LUMO_{2}|LUMO_{1}\;LUMO_{2}\rangle$$

This way, in fact, due the inter-orbital interactions the atomic spectra are with lifted degeneracy, i.e., the spectra employed is more on the physical hyper-spectra realm rather than on the chemical valence “frontier electronic” context. Therefore, in such circumstances, chemical reactivity and bonding should be described on locally based descriptors and no more by global energetics; such models are available with the aid of the conceptual density functional theory [97,98], yet they are beyond the frozen core approximation (which generally works for most stable chemical systems).

#### 3.3.2. Relation with Aromatic Systems

However, the frozen core orbitals three order levels HOMOs-LUMOs of Chemical Orthogonal Space may be considered in developing the aromatic treatment; that is, whereas the present approach with HOMO1 and LUMO 1 first order level employed the 2D space with orthogonal first order electronegativity and chemical hardness (Figure 2), the three such paired levels will bring the model in 3D space for electronegativity and 3D space for chemical hardness, with the same origin while having a rotation displacement one each other, standing therefore as a further “hyperbolic geometrical” generalization of the actual model of chemical reactivity and bonding The resulting formulae will account on resonance, which may correspond with common branches of two pairing/closing/intersecting hyperbolas/parabolas, thus accounting for aromaticity and having the benzene as the first paradigmatic/referential structure. Then, as here presented, one may then add successive fragments or playing around the benzene ring with various resonances (ortho-, para-, meta-), e.g., as in the quinone based systems, etc.

#### 3.3.3. Relation with Modern Valence Bond/Natural Resonance Theory

Even more, the present COS of electronegativity and chemical hardness may be employed as to providing the electronic density of valence/frontier of chemical reactivity/bonding, within the context of the so called “Chemical Field Theory: The Inverse Density Problem of Electronegativity and Chemical Hardness for Chemical Bond” [99],

(39) $$\rho_{|\chi ,\eta \rangle }\left(\varphi \left(1/r\right)\right)≅Z_{|\chi ,\eta \rangle }{}^{-1}\exp\left[-\frac{1}{k_{B}T}\;\left(-\chi \varphi^{2}+\eta \frac{\varphi^{4}}{2}\right)\right]$$

while leaving the partition function $Z_{|\chi ,\eta \rangle }$ expression to be adapted to the chemical context considered. Nevertheless, this research direction can make further connection with the modern valence bond theory [100,101] and with natural resonance theory [102,103,104], since the partition function unfolding can take into account, naturally, for all equivalent (resonance) configurations therefore. Note that, in Equation (39), the electronegativity–chemical hardness “field relationship” is of the same nature as in the present energetic global expansion of Equation (3) so assuring the chemical orthogonality space preservation. The difference with the modern valence bond/natural resonance theory appears to be in the density matrix consideration, however with a possible connection with partition function expressions by appropriate calibration of the so-called “quantitative resonance weights” on single/weak delocalization and multi/strong delocalization referential systems.

#### 3.3.4. Connection with the van der Waals Potentials

The repulsive and attraction terms driving the van der Waals potentials

(40) $$V_{vdW}\left(r\right)=C_{n}/r^{n}-C_{m}/r^{m}$$

and complexes therefore may also be “resolved” by means of electronegativity and chemical hardness, as corresponded chemical potentials, respectively through employing the electronic density Poisson equation [99,105],

(41) $$\nabla^{2}V_{vdW}\left(r\right)=-4\pi \rho_{|\chi ,\eta \rangle }\left(\varphi \left(1/r\right)\right)$$

to the above Equation (39); the integral solution (and by further appropriate expansion and truncation in terms of the distance (r) interaction/viz. chemical field ($\left(\varphi \left(1/r\right)\right)$) interaction) will resemble the van der Walls type (40) in the electronegativity chemical hardness chemical orthogonal space.

#### 3.3.5. Relation with the Bader’s Atoms-in Molecule’s Critical Bond

In a density-based atoms-in-molecules (AIM) model as proposed by Bader the chemical (path of) bonding is “critically” defined by the zero-flux condition of Laplacian of the electronic density [106,107],

(42) $${\left.\nabla \rho_{AIM}\left(r\right)\right|}_{R_{Bond}}=0$$

Yet, the quantum mechanical grounds of the actual model, as exposed in the present Appendix A.1 (Add-in-Bonding Chemical Scattering Paradigm) employs the potential superposition interaction under the composed form of Equations (A6) and (A7), here reported in density form as referred to the unit volume of interaction/bonding.

(43) $$\Delta v_{INTERACTION}(A,B)=\frac{\Delta \rho^{AB}}{2\Delta \mu^{AB}}\left(V_{A}^{-}V_{B}^{+}-V_{B}^{-}V_{A}^{+}\right)$$

Accordingly, once the Poisson Equation (41) is applied on last Equation (43), one has the bonding equation of AIM density

(44) $$-\rho_{AIM}\left(r\right)=\frac{\nabla \rho^{AIB}}{2\Delta \mu^{AB}}\left(V_{A}^{-}V_{B}^{+}-V_{B}^{-}V_{A}^{+}\right)+\frac{\Delta \rho^{AIB}}{2\Delta \mu^{AB}}\left(\Delta V_{A}^{-}V_{B}^{+}+V_{A}^{-}\Delta V_{B}^{+}-\Delta V_{B}^{-}V_{A}^{+}-V_{B}^{-}\Delta V_{A}^{+}\right)$$

In the case of chemical bonding Bader’s condition (43) applies on (45) so the working equation results:

(45) $$\rho_{BOND}\left(R_{Bond}\right)=\frac{C_{BOND}}{2\Delta \mu^{AB}}\Delta V_{BOND}\left(\Delta V_{B}^{-}V_{A}^{+}+V_{B}^{-}\Delta V_{A}^{+}-\Delta V_{A}^{-}V_{B}^{+}-V_{A}^{-}\Delta V_{B}^{+}\right)$$

where

(46) $$\Delta V_{BOND}={\left(\Delta V_{B}^{-}V_{A}^{+}+V_{B}^{-}\Delta V_{A}^{+}-\Delta V_{A}^{-}V_{B}^{+}-V_{A}^{-}\Delta V_{B}^{+}\right)}_{R_{Bond}}$$

Now, in the systems where the term (47) vanishes in bonding, the resulting zero density means that no-bond is predicted or formed therefore; otherwise one can further estimate the electronic density on bonding. Note that the practical application also depend on how the interaction potentials $V_{A}^{-},V_{B}^{+},V_{B}^{-},V_{A}^{+}$ are chosen—they may be further associated with resonance potentials so establishing further connection with Natural Resonance Theory too (see above and [102,103,104]). However, various chemical systems may thus be further checked by bonding formation via their adducts or molecular fragments, within the present model, besides of the various levels of computation, viz. the density functional theory and allied models, see for instance the case of bonding interaction between ortho-hydrogens in biphenyl systems [108].

## 4. Conclusions and Perspectives

The current research for establishing the structure and reactivity of molecules, macro-molecules, and even bio-molecules on the basis of their properties and functions is rich and exciting in both experimental and theoretical sides of physical chemistry. In this regard, it is worth quoting some of the preeminent and recent advancements:

- Matter-intense X-ray interaction may spectroscopically resolve the bio-molecular fragmentation and production of the high charge atomic ions aiming the bio-imaging techniques at the femtosecond X-ray regime (pump and probe). It eventually may use a synchrotron free electron laser source to investigate multiple core-ionization-Auger decays by photoionic spectroscopy [109];
- The attosecond pulses spectroscopy techniques includes the generation of X-UV light sources, X-UV + IR pulses, eventually in combination with probe-pulse and steering of moving electrons, along with photoionization time delay. They reveal the electronic dynamics in a few electrons (i.e., ionized), atoms, and molecules in various fundamental (e.g., studying the quantum wave–particle duality) and the experimental (observing or predicting fragmentation) circumstances [110];
- Visualization of photons and induced many-particles fragmentation, similar with the bubble chamber in nuclear physics, may be achieved in the eV and mili-eV regimes through the reaction microscope (imaging) techniques, e.g., Cold Target Recoil Ion Momentum Spectroscopy = COLTRIMS, along the scanning tunneling microscopes, as part of many-particle sub-atomic physics and molecular fragment dynamics [111];
- HOMO and LUMO directly relate with charge transfer occurring within the molecule, a fact established also by Fourier Transform (FT)-IR and Raman spectra. The hyperconjugation and charge delocalization models are in agreement with natural bond orbital analysis (NBO); equally, the correlations of the variational spectra with the calculated potential energy distribution (PED) and with the chemical reactivity indices (among which electronegativity and chemical hardness are preeminent measure of stability and reactivity) provide reliable results [112];
- The question of whether the electronegativity information, once inserted in the dipole derivatives and the hardness’ Hessian of molecular energy, may provide sufficient or relevant correlation or prediction with/of spectroscopic data, e.g., within IR spectroscopy, eventually through polarizable and reactive force fields, was responded in negative [113]. Therefore, electronegativity requires supplementary information as is the present chemical orthogonal space (COS), where coupling with the chemical hardness is synergistically (complementarily yet simultaneously) developed;
- Elucidation of probe mechanism of action, including reactions of metal ions and organic compounds in aqueous solutions, onto solid surfaces, and for biological activity, may be unitarily treated with the so called four-element approach of quantum chemical reactivity theory: it customarily involves (i) electronic flow driven by electronegativity, (ii) polarization by condensed local softness (i.e., the inverse of chemical hardness), (iii) electrostatic interaction by atomic partial charge exchange, and (iv) hydrophilic interaction by the inverse of apolar surface area (1/APSA) [114];
- The relationship between the Pauling difference electronegativity and energy of heteronuclear dissociations was rationalized by means of two related concepts of the electronegativity and chemical hardness/softness through a two-variable (x,y) in an absolute space of variation, thus anticipating the present COS analysis and allowing the unitary homo- and heteronuclear treatment. While “x” directly relates with Pauling electronegativity, “y” quantifies the atomic intrinsic potential, thus relating with the atomic size contribution and depending on the contributing valence orbitals, etc. [115];
- Electronegativity may correlate with the hardness of crystalline materials, such as sphalerite, wurtzite, rocksalts, oxides α-SiO_2_ and LaGaO_3_, and graphite, as well for B_12_ analogs, group IVA nitrides, tungsten carbide materials, and transition metal di- and tetra-borides. In general, the rule that similar crystal structures associate with similar hardness anisotropy is developing, while establishing that the greater bond ionicity correlates with more orderly bond arrangements in single crystals [116];
- Fragmentation of nanostructures, from polycyclic aromatic hydrocarbon molecules (PAHs) to fullerenes, highly depends on collision energies, usually ranging from few tens to few hundreds of eV, while expelling single atoms; the dominance of bonds’ fragments of C_2_- or as C_2_H_2_-molecules (or H-atoms) released from fullerenes and PAHs is recorded, respectively. However, the C_60_ clusters may feature enhanced reactivity over the van der Walls range of fullerene molecules when releasing single C-atoms by producing C_59_^+^ reactive fragments, at their turn bonding covalently with another C_60_ molecule from the remaining cluster [117];
- Detection and identification of the large ion fragmentation used in bio-imaging (e.g., for peptides) may use the gas phase coupling surface-induced dissociation (SID) in a Fourier transform ion cyclotron resonance mass spectrometer with resonant ejection of selected fragment ions using a relatively short (5 ms) ejection pulse. It is supported by the Rice–Ramsperger–Kassel–Marcus (RRKM) theory prescribing that the shape of kinetic plots follows the shape and position of the energy deposition function specific to the internal energy distribution (and thus also to the entropy) for the ion-surface collision, the deciding to (identify the) most probable reaction channels [118];
- The solid oxide fuel anode may be sensitized to produce electricity (for more than 72h), H_2_O and CO_2_ by electrochemical oxidation of CH_4_ catalyzed by the perovskite lanthanum strontium cobalt ferrite through the basic processes as (i) decomposition of CH_4_, (ii) electrochemical oxidation of H to H_2_O, and (iii) electrochemical oxidation of C to CO_2_ with the formation rate greater than that for CO [119].

Across this eminent research in the directions of electronegativity and chemical hardness, there have been distinguished as providing a special way of treating complex phenomena: by variational principles, so employing them in the most general way, they also provided a fruitful analytical tools for the quantification of atoms-in-bonding and atoms-in-molecules in a way that is not reductive to physics [120,121]. In this line, the present work succeeded in combining electronegativity and chemical hardness into the so-called chemical power, viewed as their ratio, and further inspiring the construction of the chemical orthogonal space (COS) with the consequence in generalizing the previous Parr–Pearson modelling of chemical bonding; apart of “rediscovering” the maximum charge transfer in chemical reactivity and PP approach as a limiting B→A (2D→1D) geometrical framework [6,11], the present COS also solves the long-term controversy about the non-zero values for charge transfer and exchanged energies in homonuclear bondings. In this context, it is worth mentioning that the recent discussion on dichotomy between chemical potential and electronegativity equalization principles in the light of the Wigner–Witmer symmetry correlation [122]: while the Parr–Pearson approach seems to violate it, the present COS model may accommodate it by recognizing the chemical power employs the electronegativity fluctuation (minimum) jointly with the chemical hardness (maximum) approaching the overall minimum chemical power, variational around the equilibrium of (valence to ground) states (see the diatomic applications and discussion) in bonding stabilization. it is worth mentioning the earlier [123,124,125,126] and current [127,128] alternative efforts in conceptual density functional theory providing reduction–oxidation and donor–acceptor phenomenological chemical processes as fundamental paradigms of chemical bonding and reactivity, which may be further connected with the present approach, via “chemical power,” by various expressions of “redox reorganization energies” and “donor–acceptor coherent charge transfer,” respectively.

Another distinctive aspect of the report is the present changing in the paradigm of COS vs. Parr–Pearson, being as apparent that now the role of “*A* or *B* species’ potential” is played in fact by the scattering coupling (chemical reactivity) potentials, $\eta_{A}\chi_{B}$ and $\eta_{B}\chi_{A}$, i.e., electronegativity, the reactive propensity of one species with chemical hardness –inertia of reactivity of the other species; this way, the difference in the mixing scattering products in Equation (18) $\eta_{A}\chi_{B}-\eta_{B}\chi_{A}$ effectively spans all mixing influences of species being in their turn reactive (inert) to other and inert (reactive) to own species, with the “wining” direction decided by which product of own-reactivity-coupled-with-adduct-inertia is bigger than the inverse situation own-inertia-coupled-with-adduct-reactivity. The resulting picture is more complex that the earlier one of Parr–Pearson, yet it is very physical too, while having a plus-value in modeling reactivity involving chemical inertia (chemical hardness) besides electronegativity/chemical potential alone in numerators of charge transfer. In quantum mechanical terms (see also [15,16]), the present approach considers both creation by electronegativity and annihilation by chemical hardness processes in igniting the electronic charge transfer as a cause for the chemical reactivity of the interacting adducts (see also Appendix A.1).

Moreover, the actual COS approach allows the recursive add-atoms and add-bondings patterning of poly-atomic molecular formations or with solid state add-atomic fragments interaction through channels obeying the variational selection based on the chemical power minimum principle (for its relation through the charge transfer in COS vs. PP verification of chemical reactivity by hard and soft acids and bases reactivity principle see Appendix A.2). Further experimental cross-validations of the present conceptual–numerical findings are expected in the years to come by techniques—some of them here quoted—unveiling and exploring the structure and reactivity of atoms in molecules in real time within the new field of nanochemistry.

## Figures and Tables

**Table 1:** The comparison between sensitivity indices (charge transfer, average chemical hardness, average electronegativity and exchanged energy, from the top to the bottom, respectively) for Parr–Pearson and present COS approaches for the main cases of chemical bonds for diatomic molecules: with hetero- and homo- atomic constituents, as provided by Section 2.2 and Section 2.3, respectively.

Bond Type		Parr–Pearson	COS
*AB*	$\Delta N_{AB}$	$\frac{\chi_{B}-\chi_{A}}{2(\eta_{A}+\eta_{B})}$	$\frac{1}{2}\left(\frac{\chi_{B}}{\eta_{B}}-\frac{\chi_{A}}{\eta_{A}}\right)$ $=\frac{\chi_{B}\eta_{A}-\chi_{A}\eta_{B}}{2\eta_{A}\eta_{B}}$
	$\eta_{AB}$	$2\frac{\eta_{A}\eta_{B}}{\eta_{A}+\eta_{B}}$	$\frac{\chi_{A}(\eta_{B}-\eta_{A})-\eta_{A}(\chi_{B}-\chi_{A})}{\left(\eta_{B}-\eta_{A})\tan\left\{\frac{1}{2}\left[\arctan\left(\frac{\chi_{A}}{\eta_{A}}\right)+\arctan\left(\frac{\chi_{B}}{\eta_{B}}\right)\right]\right\}-(\chi_{B}-\chi_{A}\right)}$
	$\chi_{AB}$	$\frac{\eta_{A}\chi_{B}+\eta_{B}\chi_{A}}{\eta_{A}+\eta_{B}}$	$\eta_{AB}\tan\left\{\frac{1}{2}\left[\arctan\left(\frac{\chi_{A}}{\eta_{A}}\right)+\arctan\left(\frac{\chi_{B}}{\eta_{B}}\right)\right]\right\}$
	$\Delta E_{AB}$	$-\frac{1}{2}(\chi_{B}-\chi_{A})\Delta N$	$\frac{1}{2}\left(-\chi_{AB}\Delta N_{AB}+\eta_{AB}\Delta N_{AB}^{2}\right)$
*AA*	$\Delta N_{AA}$	0	$\frac{\chi_{A}}{2\eta_{A}}-\frac{1}{2}\tan\left\{\frac{1}{2}\left[\arctan\left(\frac{-\eta_{A}+\sqrt{\eta_{A}^{2}+\chi_{A}^{2}}}{\chi_{A}}\right)+\arctan\left(\frac{\chi_{A}+\sqrt{\eta_{A}^{2}+\chi_{A}^{2}}}{\eta_{A}}\right)\right]\right\}$
	$\eta_{AA}$	$\eta_{A}$	$\frac{\eta_{A}^{2}+\chi_{A}^{2}}{\eta_{A}+\chi_{A}\tan\left[\frac{1}{2}\arctan\left(\frac{\chi_{A}}{\eta_{A}}\right)+\frac{1}{4}\arctan\left(\frac{-\eta_{A}+\sqrt{\eta_{A}^{2}+\chi_{A}^{2}}}{\chi_{A}}\right)+\frac{1}{4}\arctan\left(\frac{\chi_{A}+\sqrt{\eta_{A}^{2}+\chi_{A}^{2}}}{\eta_{A}}\right)\right]}$
	$\chi_{AA}$	$\chi_{A}$	$\eta_{AA}\tan\left[\frac{1}{2}\arctan\left(\frac{\chi_{A}}{\eta_{A}}\right)+\frac{1}{4}\arctan\left(\frac{-\eta_{A}+\sqrt{\eta_{A}^{2}+\chi_{A}^{2}}}{\chi_{A}}\right)+\frac{1}{4}\arctan\left(\frac{\chi_{A}+\sqrt{\eta_{A}^{2}+\chi_{A}^{2}}}{\eta_{A}}\right)\right]$
	$\Delta E_{AA}$	0	$\frac{1}{2}\left(-\chi_{AA}\Delta N_{AA}+\eta_{AA}\Delta N_{AA}^{2}\right)$

**Table 2:** The numerical comparison between sensitivity indices (charge transfer, average hardness, average electronegativity, exchanged energy and the introduced chemical power) for Parr–Pearson (in table: PP) and present (in table: COS) approaches for selected diatomic molecules, respectively. *Δ_f_H* is the gas phase standard enthalpy of formation at 298.15 K [75]. All energetic values are in eV (electron-volts) [76].

AB	*Δ_f_H* (eV)	η ^a^		χ ^a^		$\Delta N_{AB}$		$\eta_{AB}$		$\chi_{AB}$		$\Delta E_{AB}\;(\mathrm{eV})$		$C_{\pi }(AB)$	
		$\eta_{A}$	$\eta_{B}$	$\chi_{A}$	$\chi_{B}$	PP	COS	PP	COS	PP	COS	PP	COS	PP	COS
HF	−2.833	6.42	7.01	7.17	10.41	0.121	0.184	6.70	6.68	8.71	8.58	−0.195	−0.676	0.65	0.643
IF	−0.992	3.69	7.01	6.76	10.41	0.171	−0.173	4.83	4.95	8.02	8.15	−0.311	0.781	0.829	0.823
BrF	−0.972	4.22	7.01	7.59	10.41	0.126	−0.157	5.27	5.36	8.65	8.74	−0.177	0.751	0.821	0.815
HCl	−0.957	6.42	4.68	7.17	8.30	0.051	0.328	5.41	5.55	7.82	7.74	−0.029	−0.971	0.723	0.698
ClF	−0.521	4.68	7.01	8.30	10.41	0.09	−0.144	5.61	5.69	9.14	9.21	−0.01	0.723	0.815	0.810
HBr	−0.376	6.42	4.22	7.17	7.59	0.02	0.341	5.09	5.26	7.42	7.39	−0.004	−0.954	0.729	0.702
BrCl	0.151	4.22	4.68	7.59	8.30	0.04	−0.013	4.44	4.44	7.93	7.93	−0.014	0.05	0.893	0.893
ICl	0.184	3.69	4.68	6.76	8.30	0.092	−0.029	4.13	4.13	7.44	7.45	−0.071	0.111	0.901	0.901
HI	0.275	6.42	3.69	7.17	6.76	−0.02	0.358	4.69	4.9	6.91	6.94	−0.004	−0.928	0.737	0.708
IBr	0.423	3.69	4.22	6.76	7.59	0.052	−0.017	3.94	3.94	7.15	7.15	−0.022	0.061	0.908	0.908

^a^ from Ref. [1,2,5].

**Table 3:** The same type of data as in Table 2, but for alkaline hydrides and salts as polar molecules in a crystal state.

*AB*	*Δ_f_H*^a^ (eV)	η ^b^		χ ^b^		$\Delta N_{AB}$		$\eta_{AB}$		$\chi_{AB}$		$\Delta E_{AB}$		$C_{\pi }(AB)$	
		$\eta_{A}$	$\eta_{B}$	$\chi_{A}$	$\chi_{B}$	PP	COS	PP	COS	PP	COS	PP	COS	PP	P
LiF	−6.384	2.39	7.01	3.01	10.41	0.394	0.113	3.56	3.47	4.89	4.74	−1.456	−0.246	0.686	0.683
NaF	−5.976	2.30	7.01	2.85	10.41	0.406	0.123	3.46	3.36	4.72	4.56	−1.535	−0.255	0.681	0.677
NaCl	−4.262	2.30	4.68	2.85	8.30	0.39	0.267	3.08	2.96	4.65	4.36	−1.064	−0.477	0.753	0.737
LiCl	−4.235	2.39	4.68	3.01	8.30	0.374	0.257	3.16	3.05	4.8	4.53	−0.99	−0.482	0.758	0.743
NaBr	−3.743	2.30	4.22	2.85	7.59	0.363	0.28	2.98	2.87	4.52	4.26	−0.861	−0.483	0.759	0.742
LiBr	−3.640	2.39	4.22	3.01	7.59	0.346	0.27	3.05	2.95	4.67	4.42	−0.793	−0.488	0.764	0.748
NaI	−2.983	2.30	3.69	2.85	6.76	0.326	0.296	2.83	2.75	4.35	4.11	−0.638	−0.488	0.768	0.748
LiI	−2.803	2.39	3.69	3.01	6.76	0.308	0.286	2.9	2.82	4.48	4.26	−0.578	−0.494	0.773	0.754
LiH	−0.938	2.39	6.42	3.01	7.17	0.236	−0.071	3.48	3.54	4.14	4.2	−0.491	0.159	0.594	0.593
NaH	−0.584	2.30	6.42	2.85	7.17	0.248	−0.061	3.39	3.44	3.99	4.04	−0.535	0.13	0.589	0.588

^a^ from references [75,76]; ^b^ from references [1,2,5].

**Table 4:** The numerical comparison between sensitivity indices (charge transfer, average hardness, average electronegativity, exchanged energy, and the introduced chemical power) for Parr–Pearson (in table: PP) and present chemical orthogonal space (in table: COS) approaches for selected diatomic molecules formed by homo-atoms; enthalpy of formation *Δ*H, the enthalpy difference *ΔΔ*H = H(298.15 K) − H(0) and the thermodynamic entropy were used as experimental counterpart. All energies are in eV (electron-volts).

A_2_	S (J∙K^−1^∙mol^−1^) ^a,b^ (eV)	*Δ*_f_H ^b^ (eV)	*ΔΔ*H ^b^ (eV)	Indices ^b^ (eV)		*ΔN_AA_*		*η_AA_* (eV)		*χ_AA_* (eV)		*E_AA_* (eV)		*C_π_*(*AA*)	
				*η_A_*	*χ_A_*	PP	COS	PP	COS	PP	COS	PP	COS	PP	COS
Cl_2_ (g)	0.689	0	0.095	4.68	8.30	0	0.228	4.68	5.25	8.30	7.98	0	−0.774	0.887	0.761
Br_2_ (g)	0.756	0.32	0.101	4.22	7.59	0	0.237	4.22	4.75	7.59	7.3	0	−0.73	0.889	0.768
I_2_ (g)	0.806	0.647	0.105	3.69	6.76	0	0.248	3.69	4.17	6.76	6.5	0	−0.677	0.916	0.778
F_2_ (g)	0.627	0	0.091	7.01	10.41	0	0.135	7.01	7.51	10.41	10.07	0	−0.613	0.743	0.671
O_2_ (g)	0.634	0	0.0890	6.08	7.53	0	0.063	6.08	6.28	7.53	7.37	0	−0.22	0.619	0.587
H_2_ (g)	0.404	0	0.088	6.42	7.17	0	0.03	6.42	6.52	7.17	7.08	0	−0.103	0.558	0.543
N_2_ (g)	0.592	0	0.0899	7.23	7.27	0	0.001	7.23	7.235	7.27	7.265	0	−0.005	0.503	0.502

^a^ ×[298.15 K/1000] ^b^ from references [75,76]; ^c^ from references [1,3,5].

**Table 5:** The case of H_2_O molecule: the Parr–Pearson (PP) and chemical orthogonal space (COS) reactivity information, in terms of recursively computed charge transfer, exchanged energy, chemical hardness, electronegativity, and chemical power, according to formulations given in Table 1 and of Equation (13) for various add-atoms and add-bonding combinations.

	Paths	$\Delta N^{\mathrm{PP}}$	$\Delta N^{\mathrm{COS}}$	$\eta^{\mathrm{PP}}$	$\eta^{\mathrm{COS}}$	$\chi^{\mathrm{PP}}$	$\chi^{\mathrm{COS}}$	$\Delta E^{\mathrm{PP}}$	$\Delta E^{\mathrm{COS}}$	$C_{\pi }^{\mathrm{PP}}$	$C_{\pi }^{\mathrm{COS}}$
1	H + O	0.014	0.061	6.25	6.25	7.35	7.35	−0.003	−0.212	0.589	0.588
2	(H + O)^COS^ + H	−0.007	0.03	6.33	6.34	7.26	7.26	−6.4 × 10^−4^	0.11	0.573	0.573
3	H_2_^COS^ + O	0.018	0.076	6.29	6.3	7.31	7.3	−0.004	−0.26	0.581	0.58

**Table 6:** The essential information as in Table 5, here for the target NH_3_ molecule.

	Paths	$\eta^{\mathrm{PP}}$	$\eta^{\mathrm{COS}}$	$\chi^{\mathrm{PP}}$	$\chi^{\mathrm{COS}}$	$C_{\pi }^{\mathrm{PP}}$	$C_{\pi }^{\mathrm{COS}}$
1	N + H	6.8	6.81	7.22	7.22	0.53	0.53
2	(N + H)^COS^ + H	6.61	6.61	7.19	7.19	0.544	0.544
3	N + H_2_^COS^	6.86	6.86	7.17	7.17	0.523	0.522
4	((N + H)^COS^ + H)^COS^ + H	6.51	6.51	7.18	7.18	0.551	0.551
5	(N + H)^COS^ + H_2_^COS^	6.66	6.66	7.15	7.15	0.536	0.536
6	(N + H_2_^COS^)^COS^ + H	6.63	6.64	7.17	7.17	0.541	0.540

**Table 7:** The same information as in Table 6 for the target CH_4_ molecule.

No.	Paths	$\eta^{\mathrm{PP}}$	$\eta^{\mathrm{COS}}$	$\chi^{\mathrm{PP}}$	$\chi^{\mathrm{COS}}$	$C_{\pi }^{\mathrm{PP}}$	$C_{\pi }^{\mathrm{COS}}$
1	C + H	5.62	5.65	6.66	6.68	0.593	0.592
2	(C + H)^COS^ + H	6.01	6.02	6.91	6.91	0.574	0.574
3	C + H_2_^COS^	5.66	5.69	6.62	6.64	0.585	0.583
4	(C + H)^COS^ + H_2_^COS^	6.05	6.06	6.87	6.87	0.567	0.566
5	(C + H_2_^COS^)^COS^ + H	6.03	6.04	6.89	6.89	0.57	0.57
6	((C + H)^COS^ + H)^COS^ + H	6.21	6.22	7.04	7.04	0.566	0.566
7	((C + H)^COS^ + H)^COS^+ H_2_^COS^	6.26	6.26	6.99	6.99	0.558	0.558
8	(C + H_2_^COS^)^COS^+ H_2_^COS^	6.07	6.08	6.85	6.85	0.563	0.562
9	((C + H)^COS^ + H_2_^COS^)^COS^ + H	6.23	6.23	7.02	7.02	0.562	0.562
10	((C + H_2_^COS^)^COS^ + H)^COS^ + H	6.22	6.22	7.03	7.03	0.564	0.564
11	(((C + H)^COS^ + H)^COS^ + H)^COS^ + H	6.31	6.31	7.1	7.1	0.562	0.562

**Table 8:** The same information as in Table 6 and Table 7 for the target C_6_H_6_ molecule; the final add-bonding (+…) indicates the necessary closure step in benzene ring formation.

	Paths	$\eta^{\mathrm{PP}\text{-}P}$	$\eta^{\mathrm{COS}}$	$\chi^{\mathrm{PP}\text{-}P}$	$\chi^{\mathrm{COS}}$	$C_{\pi }^{\mathrm{PP}}$	$C_{\pi }^{\mathrm{COS}}$
1	CH + CH + CH + CH + CH + CH (+CH)	5.65	5.65	6.68	6.68	0.590 (362)	0.590 (361)
2	CH + CH + C_2_H_2_ + C_2_H_2_ (+CH)	5.74 (394)	5.74 (552)	6.6	6.6	0.574 (417)	0.574 (14)
3	CH + C_2_H_2_ + C_2_H_2_ + CH (+CH)	5.68	5.68	6.66	6.66	0.586 (456)	0.586 (435)
4	CH + C_2_H_2_ + CH + C_2_H_2_ (+CH)	5.69	5.69	6.42	6.42	0.583 (375)	0.583 (315)
5	CH + C_2_H_2_ + C_3_H_3_ (+CH)	5.74 (343)	5.74 (498)	6.6	6.6	0.574 (685)	0.574 (416)
6	C_2_H_2_ + C_2_H_2_ + C_2_H_2_ (+C_2_H_2_)	5.81 (542)	5.81 (553)	6.54	6.54	0.562 (089)	0.562 (07)
7	C_2_H_2_ + CH + CH + C_2_H_2_ (+C_2_H_2_)	5.77 (245)	5.77 (25)	6.58	6.58	0.571 (047)	0.571 (026)
8	C_2_H_2_ + C_3_H_3_ + CH (+C_2_H_2_)	5.74 (592)	5.74 (625)	6.6	6.6	0.574 (128)	0.574 (07)
9	C_2_H_2_ + C_2_H_2_ + CH + CH (+C_2_H_2_)	5.74 (999)	5.75 (026)	6.59	6.59	0.573 (389)	0.573 (342)
10	C_2_H_2_ + CH + C_3_H_3_ (+C_2_H_2_)	5.81	5.82	6.54	6.54	0.562 (357)	0.562 (34)
11	C_3_H_3_ + C_3_H_3_ (+C_3_H_3_)	5.78	5.78	6.57	6.57	0.568 (445)	0.568 (342)
12	C_3_H_3_ + C_2_H_2_ + CH (+C_3_H_3_)	5.74	5.74	6.61	6.61	0.580 (217)	0.580 (214)
13	C_3_H_3_ + CH + C_2_H_2_ (+C_3_H_3_)	5.73	5.73	6.61	6.61	0.577 (135)	0.577 (133)
14	C_3_H_3_ + CH + CH + CH (+C_3_H_3_)	5.69	5.69	6.65	6.65	0.584 (122)	0.584 (093)

**Figure A1:**
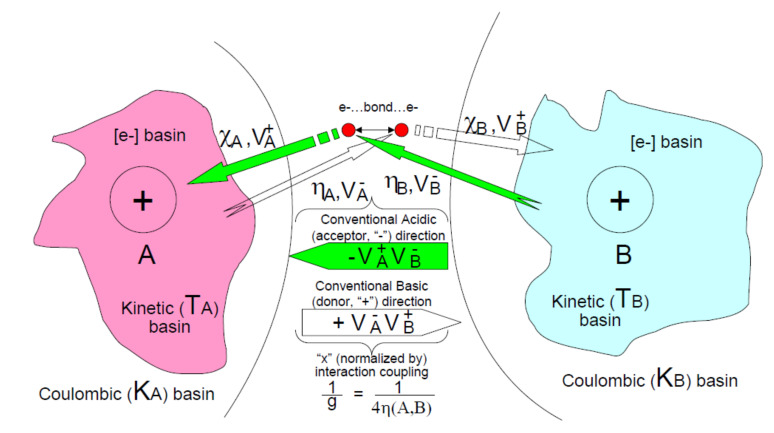
Adds-in-bonding modeling by normalized chemical reactivity scattering picture; see text for further explanations.

**Figure A2:**
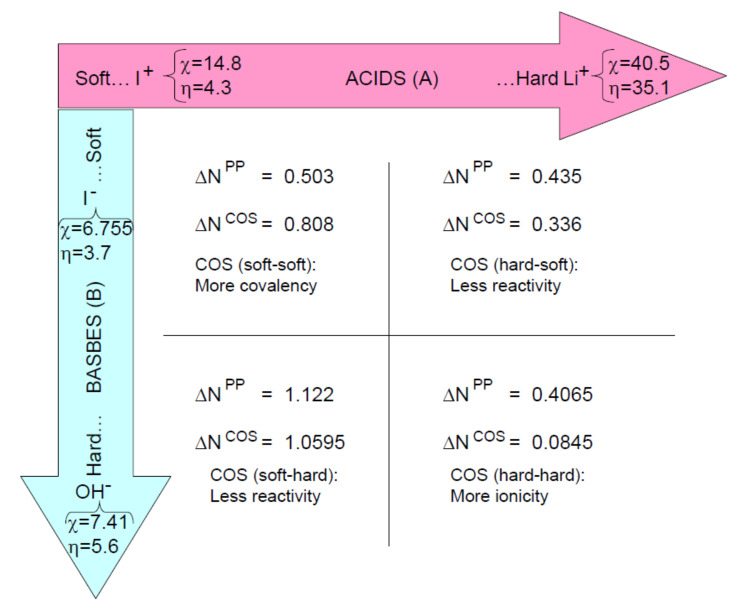
Hard and soft acids and bases (HSAB) principle’s verification matrix for Parr–Pearson (PP) vs. Chemical Orthogonal Space (COS) paradigms, i.e., by employing the absolute output of Equations (8) and (18), respectively. The electronegativities and chemical hardness values are in electron-volts (eV), see Ref. [5].

## Appendix A. Quantum-Like Physical Formalization and Chemical Implications of COS

More details on physical formalization of charge transfer as appeared in Chemical Orthogonal Space (COS) are given in Appendix A.1. To this aim, the chemical bonding formation in the formal framework of scattering (quantum) theory refers to chemical reactivity, i.e., by accounting the chemical “potentials” (electronegativity and chemical hardness), thus influencing in driving fluxes of electrons between two adjacent compounds. This picture is consistent also with the acids (*A*)–bases (*B*) Lewis interaction, whose numerical illustration and comparing with earlier Parr–Pearson (PP) formulation, Equation (8), is given in Appendix A.2, as a verification of the consecrated Hard and Soft Acids and Bases (HSAB) chemical reactivity principle [25,35,41].

### Appendix A.1. Add-in-Bonding Chemical Scattering Paradigm

Phenomenological, when atoms approach each other to form a chemical bond, they interact principally in the same manner as two quantum systems interact; however, the complication arises from the fact their interaction is both by their nuclei and electronic basins, in a very complex manner combining attraction and repulsion forces; they are nevertheless converted in manifested or virtual fluxes of charges from one adduct to the other, either they are of acidic (with fundamental accepting) or basic (with fundamental donating) electronic nature. As a consequence, a one-to-one scattering (perturbation) quantum physical theory to chemical bonding formation is not directly possible unless adapted to the specific chemical reactivity influences of the chemical bonding features; they refer to orthogonal, here in the sense of complementary/independent, quantum-like creation/annihilation actions as coming from

- electronegativity (*χ*), as a measure of attraction of electrons to a system (and associating therefore with “creation action: ${•}^{+}|A/B\rangle$”, of a particle/electron by such effect), and to
- chemical hardness (*η*), measuring the inertia/resistance of a chemical system to the reactivity (so associating with the “annihilation action: ${•}^{-}|A/B\rangle$” process for a charge transfer effect).

Of course, besides manifesting the “observables/eigen-values” for the chemical reactivity indices *χ*&*η*, the creation/annihilation actions change also the initial chemical state $|A/B\rangle$ into its complementary one $|B/A\rangle$

(A1) $$\begin{array}{l} {•}^{+}|A/B\rangle =\chi_{A/B}|B/A\rangle \\ {•}^{-}|A/B\rangle =\eta_{A/B}|B/A\rangle \end{array}$$

This is possible since the simplified quantum space of chemical reactivity encompasses only two states ($\left\{|A\rangle ,|B\rangle \right\}$), collectively associated to adducts’ electronic basins (see Figure A1).

On the other side, the chemical description of the A-B bonding is considering by following paradigmatic Lewis acids and bases gauge transformation

(A2) A+:B→A:B

However, as in valence bond theory, one should take into account for the 4 possibilities, in the electrons’ assignment in such a bond, by the associate (Pauling) resonance channels, see Ref. [3] (Vol. 3).

(A3) $$A+:B\to \left\{\begin{array}{l} A(1e^{-})-(1^{\prime }e^{-})B\\ A(1^{\prime }e^{-})-(1e^{-})B\\ A^{-}(2e^{-}):B^{+}\\ A^{+}:(2e^{-})B^{-} \end{array}\right.$$

These four alternative (Pauling virtual/resonance) channels are to be collected in the so called “interaction coupling” that normalizes/modulates the A–B reciprocal potentials’ influence in accepting/donating electrons through different reactivity mechanisms in bonding (Figure A1).

- The system A may attract an electron from bonding by $V_{A}^{+}$ potential; at the same time, the system A may resist (“refuse”) the second electron from bonding since it repels with the first electron in bonding, through the repulsive action potential $V_{A}^{-}$;
- The same is valid also for the system B, developing the potentials $V_{B}^{+}\&V_{B}^{-}$ for attracting one electron from bonding and repelling to the other, respectively.

The overall picture of scattering chemical reactivity should mix all these influences by transforming the adducts’ states, one into other, through the electronegativity and chemical hardness (chemical reactivity indices, aka eigen-values for the corresponding potentials), following the above quantum-like rules (A1), respectively, as

(A4) $$\begin{array}{l} V_{A}^{+}|\psi_{A}\rangle =\chi_{A}|\psi_{B}\rangle \\ V_{A}^{-}|\psi_{A}\rangle =\eta_{A}|\psi_{B}\rangle \\ V_{B}^{+}|\psi_{B}\rangle =\chi_{B}|\psi_{A}\rangle \\ V_{B}^{-}|\psi_{B}\rangle =\eta_{B}|\psi_{A}\rangle \\ \ldots \end{array}$$

We may proceed with the energetic contribution in bonding formation by considering the general Hamiltonian, while identifying the universal Hohenberg–Kohn functional part (cumulating the kinetic and Coulombic contributions), see Ref. [3] (Vol. 1):

(A5) $$H=\underset{F_{Hohenberg-Kohn}}{\underset{︸}{T[\rho ]+K[\rho ]}}+\underset{\begin{array}{c} CHEMICAL\;\;\;REACTIVITY\\ \left(including\;\;\;echange-correlation\right) \end{array}}{\underset{︸}{V_{INTERACTION}(\chi [\rho ],\eta [\rho ])}}$$

In Equation (A5), the interaction potential is driving the chemical reactivity and the above reactivity potentials and creating/annihilating, viz. accepting/donating states in a mixed manner by overlapping the acidic and basic directions of charge transfer fluxes (with their coupling signs) as represented in the Figure A1. However, in “chemical scattering” the chemical reactivity excess/stabilizing energy of bonding appears just from the adducts’ interaction, by their potentials’ superposition, that is,

(A6) $$\Delta H=\Delta V_{INTERACTION}(A,B)=\frac{1}{4\eta (A,B)}\left(V_{A}^{-}V_{B}^{+}-V_{B}^{-}V_{A}^{+}\right)$$

The denominator was identified as the anticipated coupling factor of A-B bonding:

- it normalizes the mixed potentials of chemical reactivity, so it appears in the denominator;
- it is written as four times the resistance of the mixed contribution to the bonding-by-reactivity potential (see above the Pauling resonance argument);
- such mixed resistance to bonding/reactivity corresponds to the chemical hardness kernel.

Nevertheless, the current hardness reactivity kernel is considered in the so called “covariant” definition of AB–bonding:

(A7) $$\eta (A,B)=\frac{1}{2}\frac{\Delta \mu^{BA}}{\Delta N^{BA}}$$

“Covariancy” is a physical term that accounts for “transitivity effect”; it is extended to chemical species as following: if “*BA*” represents “*B* has the interaction (electronic charge transfer) flux oriented to *A*,” then the simultaneously appearance of “*BA*” to numerator and “*BA*” to denominator resulted in a two-origin phenomenon (*BA*…*BA* = *BA*, so linking *B* with *A*); instead, having the situation “*BA*” in combination with “*AB*” of interaction fluxes equivalents with a “loop” (*BA*…*AB* = *BB* or *AB*…*BA* = *AA*) with a single-origin phenomenon B or *A*, depending which from *BA* and *AB* is on numerator and denominator, driving/triggering the interaction, respectively. This way, the single-system chemical hardness will be abstracted from the Equation (A7) with the following specific definition:

(A8) $$\eta (A)=\frac{\Delta \mu^{AB}}{\Delta N^{BA}},\;\eta (B)=\frac{\Delta \mu^{BA}}{\Delta N^{AB}}$$

In Equation (A8), the (1/2) pre-factor of Equation (A7) was dropped for having a closed flux circuit with single origin, A or B, respectively.

When we go to the observability level of Equation (A6), the quantum-like interaction potential is considered as an observable, with the form

(A9) $$\langle \psi_{A}|\Delta V_{INTERACTION}(A,B)|\psi_{B}\rangle =\Delta \mu^{AB}\langle \psi_{A}|\psi_{B}\rangle$$

Note that the Equation (A9) relates the stabilization energy in bonding interaction with the chemical potential flux $\Delta \mu^{AB}$; however, here, it is modulated by the overlap matrix (or integral $\langle \psi_{A}|\psi_{B}\rangle$) of adduct states in bonding. This is nevertheless an important link with the previous Parr–Pearson approach for the charge transfer formulation, Equation (8), at it is turn being driven by the difference of adducts’ chemical potential (or electronegativity with changed sign), yet here with more complex (quantum-like) physical construction. Applying the quantum observability (bra-ket) product for the adduct A-to-B states to the entire Equation (A6), we thus have

(A10) $$\langle \psi_{A}|\Delta V_{INTERACTION}(A,B)|\psi_{B}\rangle =\frac{\langle \psi_{A}|V_{A}^{-}V_{B}^{+}-V_{B}^{-}V_{A}^{+}|\psi_{B}\rangle }{4\eta (A,B)}$$

Equation (A10) rewrites by the quantum-like rules (A4) and (A9) to become

(A11) $$\Delta \mu^{AB}\langle \psi_{A}|\psi_{B}\rangle =\frac{\eta_{A}\chi_{B}-\eta_{B}\chi_{A}}{4\eta (A,B)}\langle \psi_{A}|\psi_{B}\rangle$$

Finally, Equation (A11) may be appropriately rearranged through employing the definitions for the covariant kernel (A7) and the single chemical hardness (A8) to leave with the working result

(A12) $$\Delta N^{AB}=\frac{\eta_{A}\chi_{B}-\eta_{B}\chi_{A}}{2\left(\frac{\Delta \mu^{AB}}{\Delta N^{BA}}\right)\left(\frac{\Delta \mu^{BA}}{\Delta N^{AB}}\right)}=\frac{\eta_{A}\chi_{B}-\eta_{B}\chi_{A}}{2\eta_{A}\eta_{B}}$$

The last equation recovers the basic electronic charge transfer formulation derived within chemical orthogonal space (COS), Equation (18), yet here re-developed on more elaborated assumptions involving scattering chemical reactivity picture. An operational verification of the reliability of the present charge transfer formulation by Equation (A12) aka (18) vs. Parr–Pearson Equation (8) is given in Appendix A.2 for one of the most celebrated principle of chemical reactivity.

### Appendix A.2. Hard-and-Soft-Acids-and-Bases (HSAB) Principle’s Verification

According with Pearson, the classification of acids and bases as hard and soft combines, apart from inherent chemical hardness high/low values, the electronegativity influence, precisely as [2,25,35]:

- a soft base, e.g., R^−^ or H^−^, is very polarizable and thus with low electronegativity;
- a hard base, e.g., OH^−^, is not much polarizable and thus with high electronegativity (respecting the preceding soft base case);
- a soft acid, e.g., RO^+^ or HO^+^, has usually low positive charge and large size, so posing lower electronegativity;
- a hard acid, e.g., H^+^ or XH (hydrogen bonding molecules), has normally high positive charge and small size, so featuring high electronegativity (respecting the preceding soft acid case).

Having these operational definitions/classifications of chemical compounds, a wide chemical reactions can be rationalized on the principle according which “soft acids like soft bases” (s-s), “hard acids like hard bases” (h-h), and “soft (hard) acids do not like hard (soft) bases” (s-h, h-s)—so consecrating the Hard and Soft Acids and Bases (HSAB) principle of chemical reactivity. Parr and Pearson already lucidly analyzed these cases and find respectively that, see Ref. [5]:

- The (s-s) case corresponds to the covalent bonding case and requires higher charge transfer (ΔN↑);
- The (h-h) case, paradoxically, does not furnishes high charge transfer (ΔN↓), yet being this way consistent with ionicity character of bonding by employing the same operational definition for ΔN;
- The (s-h) and (h-s) cases should not favor chemical reactivity by lowering charge transferred quantities (ΔN↓).

Accordingly, in the Figure A2 the HSAB verification matrix was provided for representative soft and hard acids and bases, for which both Parr–Pearson and actual Chemical Orthogonal Space operational formulations for charge transfer evaluations, Equations (8) and (18), were respectively applied. Remarkably, for each combination, the actual COS approach provides better charge transfer environment for characterization of the chemical bonding and chemical reactivity as consecrated by Parr and Pearson themselves, namely,

- For (s-s) case: $\Delta N^{PP}<\Delta N^{COS}$ meaning that COS is more appropriate for covalency characterization;
- For (h-h) case: $\Delta N^{PP}>\Delta N^{COS}$ resulting COS as better description for ionicity character;
- For (s-h) and (h-s) cases: $\Delta N^{PP}>\Delta N^{COS}$ so affirming COS as less allowing the chemical reactivity by charge transfer, as the general prescription indicates.

All in all, the present HSAB verification, besides the quantum-like physical formalization exposed in Appendix A.1, may give confidence the present Chemical Orthogonal Space and may be considered as both generalizing and improving the previous, otherwise very instructive, approach of Parr–Pearson for add-in-bonding interaction. Further theoretical developments, conceptual interpretations, and numerical applications are given in the main text (Section 2.3, Section 2.4, Section 3.1 and Section 3.2).

## References

[1] Parr R.G., Yang W. (1989). Density Functional Theory of Atoms and Molecules.

[2] Pearson R.G. (1997). Chemical Hardness.

[3] Putz M.V. (2016). Quantum Nanochemistry. A Fully Integrated Approach (5 Volumes Package): Vol. 1. Quantum Theory and Observability; Vol. 2. Quantum Atoms and Periodicity; Vol. 3. Quantum Molecules and Reactivity; Vol. 4. Quantum Solids and Orderability; Vol. 5. Quantum Structure-Activity Relationship (Qu-SAR).

[4] Parr R.G., Donnelly R.A., Levy M., Palke W.E. (1978). Electronegativity: The density functional viewpoint. J. Chem. Phys..

[5] Parr R.G., Pearson R.G. (1983). Absolute hardness: Companion parameter to absolute electronegativity. J. Am. Chem. Soc..

[6] Parr R.G., Szentpály L.V., Liu S. (1999). Electrophilicity index. J. Am. Chem. Soc..

[7] Putz M.V., Putz M.V. (2011). Quantum and electrodynamic versatility of electronegativity and chemical hardness. Quantum Frontiers of Atoms and Molecules.

[8] Putz M.V. (2008). Density functionals of chemical bonding. Int. J. Mol. Sci..

[9] Chattaraj P.K., Lee H., Parr R.G. (1991). Principle of maximum hardness. J. Am. Chem. Soc..

[10] Chattaraj P.K., Liu G.H., Parr R.G. (1995). The maximum hardness principle in the Gyftpoulos-Hatsopoulos three-level model for an atomic or molecular species and its positive and negative ions. Chem. Phys. Lett..

[11] Parr R.G., Bartolotti L.J. (1982). On the geometric mean principle of electronegativity equalization. J. Am. Chem. Soc..

[12] Parr R.G., Bartolotti L.J. (1983). Some remarks on the density functional theory of few-electron systems. J. Phys. Chem..

[13] Bergmann D., Hinze J. (1987). Electronegativity and charge distribution. Struct. Bond..

[14] Mulliken R.S. (1934). A new electroaffinity scale: Together with data on valence states and an ionization potential and electron affinities. J. Chem. Phys..

[15] Putz M.V. (2009). Electronegativity: Quantum observable. Int. J. Qual. Chem..

[16] Putz M.V. (2010). Chemical Hardness: Quantum Observable?. Studia Universitatis Babeş-Bolyai—Seria Chemia–Tom I.

[17] Koopmans T. (1934). Uber die Zuordnung von Wellen Funktionen und Eigenwerter zu den Einzelnen Elektronen Eines Atom. Physica.

[18] Putz M.V. (2013). Koopmans’ analysis of chemical hardness with spectral like resolution. Sci. World J..

[19] Komorowski L. (1987). Electronegativity and hardness in chemical approximation. Chem. Phys..

[20] Putz M.V. (2006). Systematic formulation for electronegativity and hardness and their atomic scales within density functional softness theory. Int. J. Quantum Chem..

[21] Ayers P.W., Parr R.G. (2000). Variational principles for describing chemical reactions: The Fukui function and chemical hardness revisited. J. Am. Chem. Soc..

[22] Ayers P.W., Parr R.G. (2001). Variational principles for describing chemical reactions: Reactivity indices based on the external potential. J. Am. Chem. Soc..

[23] Sanderson R.T. (1988). Principles of electronegativity Part I. General nature. J. Chem. Educ..

[24] Tachibana A., Nakamura K., Sakata K., Morisaki T. (1999). Application of the regional density functional theory: The chemical potential inequality in the HeH+ system. Int. J. Quantum Chem..

[25] Pearson R.G. (1990). Hard and soft acids and bases—The evolution of a chemical concept. Coord. Chem. Rev..

[26] Chattaraj P.K., Sarkar U., Roy D.R. (2007). Electronic structure principles and aromaticity. J. Chem. Edu..

[27] Putz M.V. (2011). Chemical action concept and principle. MATCH Commun. Math. Comput. Chem..

[28] Ghanty T.K., Ghosh S.K. (1996). New scale of atomic orbital radii and its relationships with polarizability, electronegativity, other atomic properties, and bond energies of diatomic molecules. J. Phys. Chem..

[29] Robles J., Bartolotti L.J. (1984). Electronegativities, electron affinities, ionization potentials, and hardnesses of the elements within spin polarized density functional theory. J. Am. Chem. Soc..

[30] Chattaraj P.K., Duley S. (2010). Electron affinity, electronegativity, and electrophilicity of atoms and ions. J. Chem. Eng. Data.

[31] Dauben H.J., Wilson J.D., Laity J.L. (1968). Diamagnetic susceptibility exaltation as a criterion of aromaticity. J. Am. Chem. Soc..

[32] Berkowitz M., Parr R.G. (1988). Molecular hardness and softness, local hardness and softness, hardness and softness kernels, and relations among these quantities. J. Chem. Phys..

[33] Putz M.V. (2011). Electronegativity and chemical hardness: Different patterns in quantum chemistry. Curr. Phys. Chem..

[34] Putz M.V. (2010). Compactness aromaticity of atoms in molecules. Int. J. Mol. Sci..

[35] Putz M.V. (2008). Absolute and Chemical Electronegativity and Hardness.

[36] Putz M.V. (2007). Semiclassical electronegativity and chemical hardness. J. Theor. Comp. Chem..

[37] Putz M.V., Russo N., Sicilia E. (2003). Atomic radii scale and related size properties from density functional electronegativity formulation. J. Phys. Chem. A.

[38] Putz M.V., Castro E.A., Haghi A.K. (2012). Nanoroots of Quantum Chemistry: Atomic Radii, Periodic Behavior, and Bondons. Nanoscience and Advancing Computational Methods in Chemistry: Research Progress.

[39] Ghosh D.C., Islam N. (2011). Whether electronegativity and hardness manifest two different descriptors of the one and the same fundamental property of atoms—A quest. Int. J. Quantum Chem..

[40] Ghosh D.C., Islam N. (2011). Whether there is a hardness equalization principle analogous to the electronegativity equalization principle—A quest. Int. J. Quantum Chem..

[41] Putz M.V., Russo N., Sicilia E. (2004). On the application of the HSAB principle through the use of improved computational schemes for chemical hardness evaluation. J. Comp. Chem..

[42] Putz M.V. (2011). On relationship between electronic sharing in bonding and electronegativity equalization of atoms in molecules. Int. J. Chem. Model..

[43] Ciesielski A., Krygowski T.M., Cyranski M.K., Dobrowolski M.A., Balaban A.T. (2009). Are thermodynamic and kinetic stabilities correlated? A topological index of reactivity toward electrophiles used as a criterion of aromaticity of polycyclic benzenoid hydrocarbons. J. Chem. Inf. Model..

[44] Putz M.V., Putz M.V. (2011). Quantum parabolic effects of electronegativity and chemical hardness on carbon π-systems. Carbon Bonding and Structures: Advances in Physics and Chemistry.

[45] Putz M.V. (2013). Chemical orthogonal spaces (COSs): From structure to reactivity to biological activity. Int. J. Chem. Model..

[46] Putz M.V. (2013). Bonding in orthogonal space of a chemical structure: From in cerebro to in silico. Int. J. Chem. Model..

[47] Putz M.V., Ori O., Cataldo F., Putz A.M. (2013). Parabolic reactivity “coloring” molecular topology: Application to carcinogenic PAHs. Curr. Org. Chem..

[48] Tudoran M.A., Putz M.V. (2015). Molecular graph theory: From adjacency information to colored topology by chemical reactivity. Curr. Org. Chem..

[49] Gutman I., Milun M., Trinastić N. (1977). Graph theory and molecular orbitals. 19. Nonparametric resonance energies of arbitrary conjugated systems. J. Am. Chem. Soc..

[50] Putz M.V., Tudoran M.A., Ori O. (2015). Topological organic chemistry: From distance matrix to Timisoara eccentricity. Curr. Org. Chem..

[51] Putz M.V., Tudoran M.A., Mirica M.C. (2016). Quantum dots searching for bondots. Towards sustainable sensitized solar cells. Sustainable Nanosystems Development, Properties, and Applications.

[52] Putz M.V., Tudoran M.A., Mirica M.C. (2016). Bondonic electrochemistry: Basic concepts and sustainable prospects. Sustainable Nanosystems Development, Properties, and Applications.

[53] Putz M.V. (2012). Chemical Orthogonal Spaces. Mathematical Chemistry Monographs.

[54] Nalewajski R.F. (1984). Electrostatic effects in interaction between hard (soft) acids and bases. J. Am. Chem. Soc..

[55] Nalewajski R.F. (1998). Kohn-Sham description of equilibria and charge transfer in reactive systems. Int. J. Quantum Chem..

[56] Putz M.V., Putz A.M. (2013). DFT chemical reactivity driven by biological activity: Applications for the toxicological fate of chlorinated PAHs. Struct. Bond..

[57] Putz M.V., Dudaș N.A. (2013). Variational principles for mechanistic quantitative structure–activity relationship (QSAR) studies: Application on uracil derivatives’ anti-HIV action. Struct. Chem..

[58] Putz M.V., Dudaș N.A. (2013). Determining Chemical Reactivity Driving Biological Activity from SMILES Transformations: The Bonding Mechanism of Anti-HIV Pyrimidines. Molecules.

[59] Putz M.V., Tudoran M.A., Putz A.M. (2013). Structure properties and chemical-bio/ecological of pah interactions: From synthesis to cosmic spectral lines, nanochemistry, and lipophilicity-driven reactivity. Curr. Org. Chem..

[60] Kovacevic N., Kokalj A. (2011). Analysis of molecular electronic structure of imidazole- and benzimidazole-based inhibitors: A simple recipe for qualitative estimation of chemical hardness. Corros. Sci..

[61] Kaya S., Kaya C. (2015). A new equation based on ionization energies and electron affinities of atoms for calculating of group electronegativity. Comput. Theor. Chem..

[62] Guerra D., Contreras R., Perez P., Fuentealba P. (2006). Hardness and softness kernels, and related indices in the spin polarized version of density functional theory. Chem. Phys. Lett..

[63] Politzer P., Murray J.S. (2006). A link between the ionization energy ratios of an atom and its electronegativity and hardness. Chem. Phys. Lett..

[64] Meneses L., Fuentealba P., Contreras R. (2006). On the variations of electronic chemical potential and chemical hardness induced by solvent effects. Chem. Phys. Lett..

[65] Tang C., Zhu W., Deng K. (2009). The evolutions of the structure stability, vibrational frequency, frontier orbital, and electronegativity of the unconventional exohedral fullerenes C_64_X_4_ (X = H, F, Cl, Br, and I): A density functional study. J. Mol. Struct. (THEOCHEM).

[66] Matar S.F., Campet G., Subramanian M.A. (2011). Electronic properties of oxides: Chemical and theoretical approaches. Prog. Sol. State Chem..

[67] Noorizadeh S. (2005). The maximum hardness and minimum polarizability principles in accordance with the Bent rule. J. Mol. Struct. (THEOCHEM).

[68] Torrent-Sucarrat M., Luis J.M., Duran M., Sola M. (2005). An assessment of a simple hardness kernel approximation for the calculation of the global hardness in a series of Lewis acids and bases. J. Mol. Struct. (THEOCHEM).

[69] Putz M.V. (2012). Valence atom with Bohmian quantum potential: The golden ratio approach. Chem. Central J..

[70] Nataraj A., Balachandran V., Karthick T. (2013). Molecular orbital studies (hardness, chemical potential, electrophilicity, and first electron excitation), vibrational investigation and theoretical NBO analysis of 2-hydroxy-5-bromobenzaldehyde by density functional method. J. Mol. Struct..

[71] Demircioglu Z., Kastas C.A., Buyukgungor O. (2015). Theoretical analysis (NBO, NPA, Mulliken Population Method) and molecular orbital studies (hardness, chemical potential, electrophilicity and Fukui function analysis) of (E)-2-((4-hydroxy-2-methylphenylimino)methyl)-3-methoxyphenol. J. Mol. Struct..

[72] Yıldız M., Karpuz O., Zeyrek C.T., Boyacıoglu B., Dal H., Demir N., Yıldırım N., Unver H. (2015). Synthesis, biological activity, DNA binding and anion sensors, molecular structure and quantum chemical studies of a novel bidentate Schiff base derived from 3,5-bis(triflouromethyl)aniline and salicylaldehyde. J. Mol. Struct..

[73] Schroder D., Schroeter K., Zummack W., Schwarz H. (1999). Charge inversion as a structural probe for C_6_H_5_^+^ and C_6_H_6_^+.^ ations. J. Am. Soc. Mass. Spectrom..

[74] Kokalj A. (2012). On the HSAB based estimate of charge transfer between adsorbates and metal surfaces. Chem. Phys..

[75] Lide D.R., Frederikse H.P.R. (1996). CRC Handbook of Chemistry and Physics 1996–1997.

[76] Energy Units Converter. http://www.colby.edu/chemistry/PChem/Hartree.html.

[77] Light K.J., Allison J. (1990). Mechanistic considerations of the protonation and fragmentation of highly functionalized molecules in fast atom bombardment: High resolution mass spectrometry and tandem mass spectrometry analysis of the ions formed by fast atom bombardment of digoxin and related cardiac glycosides. J. Am. Soc. Mass. Spectrom..

[78] Bakhtiar R., Jacobson D.B. (1996). Transition-metal mediated heteroatom removal by reactions of FeL^+^ [L=O, C_4_H_6_,c-C_5_H_6_, c-C_5_H_5_, C_6_H_6_, C_5_H_4_(=CH_2_)] with furan, thiophene, and pyrrole in the gas phase. J. Am. Soc. Mass. Spectrom..

[79] Sobocinski P., Pesic Z.D., Hellhammer R., Stolterfoht N., Chesnel J.-Y., Legendre S., Sulik B. (2005). Fragmentation of H_2_O molecules following interaction with slow He2+ ions. Nucl. Instr. Meth. Phys. Res. B.

[80] Moreh R., Finkelstein Y., Vos M. (2015). Electron scattering as a tool to study zero-point kinetic energies of atoms in molecules. Nucl. Instr. Meth. Phys. Res. B.

[81] Park S.M., Kim M.Y., Kim E.S., Han H.S., Seo G. (2011). H_2_-SCR of NO on Pt–MnOx catalysts: Reaction path via NH_3_ formation. Appl. Catal. A Gen..

[82] Donazzi A., Livio D., Diehm C., Beretta A., Groppi G., Forzatti P. (2014). Effect of pressure in the autothermal catalytic partial oxidation of CH_4_ and C_3_H_8_: Spatially resolved temperature and composition profiles. Appl. Catal. A Gen..

[83] Kobzarenko A.V., Sukhov F.F., Orlov A.Y., Kovalev G.V., Baranova I.A., Feldman V.I. (2012). Effect of molecular structure on fragmentation of isolated organic molecules in solid rare gas matrices. Rad. Phys. Chem..

[84] Sulkes M. (2005). Selective bond fragmentation in pulsed laser ablation of ring strained molecules: Evidence for a thermal mechanism. Chem. Phys. Lett..

[85] Pillai E.D., Molek K.S., Duncan M.A. (2005). Growth and photodissociation of U^+^(C_6_H_6_)_n_ (n = 1–3) and UO^+^_m_(C_6_H_6_) (m = 1,2) complexes. Chem. Phys. Lett..

[86] Sadr-Arani L., Mignon P., Chermette H., Douki T. (2014). Theoretical and experimental study of the fragmentation of protonated uracil. Chem. Phys. Lett..

[87] Hufsky F., Scheubert K., Böcker S. (2014). Computational mass spectrometry for small-molecule fragmentation. Trend. Anal. Chem..

[88] Green F.M., Gilmore I.S., Seah M.P. (2008). G-SIMS and SMILES: Simulated fragmentation pathways for identification of complex molecules, amino acids and peptides. Appl. Surf. Sci..

[89] Green F.M., Dell E.J., Gilmore I.S., Seah M.P. (2008). Identification of complex molecules at surfaces: G-SIMS and SMILES fragmentation pathways. Int. J. Mass Spectrom..

[90] Rahemi N., Haghighi M., Babaluo A.A., Allahyari S., Jafari M.F. (2014). Syngas production from reforming of greenhouse gases CH_4_/CO_2_ over Ni–Cu/Al_2_O_3_ nanocatalyst: Impregnated vs. plasma-treated catalyst. Energ. Convers. Manag..

[91] Cimpoesu F., Zaharia A., Stamate D., Panait P., Oprea C.I., Girtu M.A., Ferbinteanu M. (2013). New insights in the bonding regime and ligand field in Wernerian complexes. A density functional study. Polyhedron.

[92] Valencia I., Tavizón G., Barba-Behrens N., Castro M. (2011). Rice-ball structures of iron–benzene clusters, Fe_4_–C_6_H_6_)_m_, m ≤ 3. A density functional study. Chem. Phys..

[93] Tamuliene J., Romanova L.G., Vukstich V.S., Snegursky A.V. (2012). Mechanisms of the electron-impact-induced glycine molecule fragmentation. Chem. Phys..

[94] Tamuliene J., Romanova L.G., Vukstich V.S., Snegursky A.V. (2012). Mechanisms of the electron-impact-induced methionine molecule fragmentation. Chem. Phys..

[95] Georgiev S., Neusser H.J. (2005). Mass analyzed threshold ionization of hydrogen bonded clusters of biological molecules: The 3-methylindole·C_6_H_6_ complex. J. Electron. Spectrosc. Relat. Phenom..

[96] Mitsuke K. (2005). Photofragmentaion mechanisms of H_2_O studied by ultraviolet dispersed spectroscopy. J. Electron. Spectrosc. Relat. Phenom..

[97] Matito E., Putz M.V. (2011). New Link between Conceptual Density Functional Theory and Electron Delocalization. J. Phys. Chem. A.

[98] Putz M.V., Chattaraj P.K. (2013). Electrophilicity Kernel and its Hierarchy through Softness in Conceptual Density Functional Theory. Int. J. Quantum Chem..

[99] Putz M.V. (2017). Chemical Field Theory: The Inverse Density Problem of Electronegativity and Chemical Hardness for Chemical Bond. Curr. Phys. Chem..

[100] Gerratt J., Cooper D.L., Karadakov P.B., Raimondi M. (1997). Modern Valence Bond Theory. Chem. Soc. Rev..

[101] Shaik S., Hiberty P.C. (2004). Valence Bond theory, its History, Fundamentals and Applications. A Primer. Rev. Comput. Chem..

[102] Glendening E.D., Weinhold F. (1998). Natural Resonance Theory: I. General Formalism. J. Comput. Chem..

[103] Glendening E.D., Weinhold F. (1998). Natural Resonance Theory: II. Natural Bond Order and Valency. J. Comp. Chem..

[104] Glendening E.D., Badenhoop J.K., Weinhold F. (1998). Natural Resonance Theory: III. Chemical Applications. J. Comp. Chem..

[105] Putz M.V., Russo N., Sicilia E. (2005). About the Mulliken Electronegativity in DFT. Theor. Chem. Acc..

[106] Bader R.F.W. (2001). The zero-flux surface and the topological and quantum definitions of an atom in a molecule. Theor. Chem. Acc..

[107] Bader R.F.W. (2009). A comment on “Some fundamental problems with zero-flux partitioning of electron densities”. *Theor. Chem. Acc.*
**2002**, *107*, 381–382; c) Bader, R.F.W. Bond paths are not chemical bonds. J. Phys. Chem. A.

[108] Poater J., Solà M., Bickelhaupt F.M. (2006). Hydrogen–Hydrogen Bonding in Planar Biphenyl, Predicted by Atoms-In-Molecules Theory, Does Not Exist. Chem. Eur. J..

[109] Berrah N., Fang L., Osipov T., Murphy B., Bostedt C., Bozek J.D. (2014). Multiphoton ionization and fragmentation of molecules with the LCLSX-ray FEL. J. Electron. Spectrosc. Relat. Phenom..

[110] Peng L.-Y., Jiang W.-C., Geng J.-W., Xiong W.-H., Gong Q. (2015). Tracing and controlling electronic dynamics in atoms and molecules by attosecond pulses. Phys. Rep..

[111] Czasch A., Schmidt L.P.H., Jahnke T., Weber T., Jagutzki O., Schössler S., Schöffler M.S., Dörner R., Schmidt-Böcking H. (2005). Photo induced multiple fragmentation of atoms and molecules: Dynamics of Coulombic many-particle systems studied with the COLTRIMS reaction microscope. Phys. Lett. A.

[112] Muthu S., Renuga S. (2014). Molecular orbital studies (hardness, chemical potential, electronegativity and electrophilicity), vibrational spectroscopic investigation and normal coordinate analysis of 5-{1-hydroxy-2-[(propan-2-yl)amino]ethyl}benzene-1,3-diol. Spectrochim. Acta Part A Mol. Biomol. Spectrosc..

[113] Verstraelen T., Bultinck P. (2015). Can the electronegativity equalization method predict spectroscopic properties?. Spectrochim. Acta Part A Mol. Biomol. Spectrosc..

[114] Chang C.M., Lin T.H., Chen Y.S., Chang C.W., Huang K.L., Wu F.W., Hsu W.J., Yu M.P., Lin C., Wang M.K. (2014). A quantum chemical approach using classical concepts to characterization and descriptive analysis of various reactions of metal ions and organic compounds. Chemometr. Intell. Lab. Syst..

[115] Smith D.W. (2007). A new approach to the relationship between bond energy and electronegativity. Polyhedron.

[116] Li K., Yang P., Xue D. (2012). Anisotropic hardness prediction of crystalline hard materials from the electronegativity. Acta Mater..

[117] Gatchell M., Stockett M.H., Rousseau P., Chen T., Kulyk K., Schmidt H.T., Chesnel J.Y., Domaracka A., Méry A., Maclot S. (2014). Non-statistical fragmentation of PAHs and fullerenes in collisionswith atoms. Int. J. Mass Spectrom..

[118] Laskin J., Futrell J.H. (2015). New approach for studying slow fragmentation kinetics in FT-ICR: Surface-induced dissociation combined with resonant ejection. Int. J. Mass Spectrom..

[119] Fisher J.C., Chuang S.S.C. (2009). Investigating the CH_4_ reaction pathway on a novel LSCF anode catalyst in the SOFC. Catal. Commun..

[120] Whitney C.K. (2008). Closing in on chemical bonds by opening up relativity theory. Int. J. Mol. Sci..

[121] Rahm M., Hoffmann R. (2015). Toward an experimental quantum chemistry: Exploring a new energy partitioning. J. Am. Chem. Soc..

[122] Von Szentpály L. (2015). Symmetry laws improve electronegativity equalization by orders of magnitude and call for a paradigm shift in conceptual density functional theory. J. Phys. Chem. A.

[123] Moens J., Geerlings P., Roos G. (2007). A conceptual DFT approach for the evaluation and interpretation of redox potentials. Chem. Eur. J..

[124] Moens J., Jaque Olmedo Pablo C., De Proft F., Geerlings P. (2008). The Study of Redoxreactions based on Conceptual DFT Principles: EEM and Vertical Quantities. J. Phys. Chem. A.

[125] Geerlings P., De Proft F., Langenaeker W. (2003). Conceptual Density Functional Theory. Chem. Rev..

[126] Maynard A.T., Huang M., Rice W.G., Covell D.G. (1998). Reactivity of the HIV-1 nucleocapsid protein p7 zinc finger domains from the perspective of density-functional theory. Proc. Natl. Acad. Sci. USA.

[127] Nalewajski R.F. (2019). Understanding Electronic Structure and Chemical Reactivity: Quantum-Information Perspective. Appl. Sci..

[128] Nalewajski R.F. (2020). Phase Equalization, Charge Transfer, Information Flows and Electron Communications in Donor–Acceptor Systems. Appl. Sci..

